# Data-driven prioritization of high-risk individuals for weight loss interventions

**DOI:** 10.1038/s41591-026-04353-2

**Published:** 2026-04-30

**Authors:** Kamil Demircan, Julia Carrasco-Zanini, Alice Williamson, Carl Beuchel, Linsey Jackson, Werner Römisch-Margl, Aleksander L. Hansen, Sarah Finer, David A. van Heel, Genes & Health Research Team, Sarah Finer, David A. van Heel, Claudia Langenberg, Gabi Kastenmüller, Matthew Coghlan, Ida Moeller, Nicholas J. Wareham, Maik Pietzner, Claudia Langenberg

**Affiliations:** 1 Precision Healthcare University Research Institute, Queen Mary University of London, London, UK; 2 Computational Medicine, Berlin Institute of Health at Charité-Universitätsmedizin Berlin, Berlin, Germany; 3 Friede Springer Cardiovascular Prevention Center at Charité-Universitätsmedizin Berlin, Berlin, Germany; 4 Eli Lilly and Company, Indianapolis, IN USA; 5 Institute of Computational Biology, Helmholtz Zentrum München - German Research Center for Environmental Health, Neuherberg, Germany; 6 Steno Diabetes Center Copenhagen, Herlev, Denmark; 7 Wolfson Institute of Population Health, Queen Mary University of London, London, UK; 8 Blizard Institute, Queen Mary University of London, London, UK; 9 IMS Epidemiology, University of Cambridge School of Clinical Medicine, Institute of Metabolic Science, Cambridge, UK; 10 Max Planck Institute for Molecular Genetics, Berlin, Germany

**Keywords:** Predictive medicine, Prognostic markers, Predictive markers

## Abstract

New obesity medications have demonstrated efficacy in trials, but their real-world deployment is partly limited by the absence of approaches that identify individuals for treatment based on risks for obesity-related complications. Here we present a risk prediction model to guide prioritization of high-risk individuals. In a population-based sample of ~200,000 individuals with a body mass index (BMI) exceeding 27 kg m^−^^2^, our machine learning framework identified the 20 most informative features, from among thousands tested, that predict future onset of 18 complications of obesity, providing information beyond BMI. An integrated model (OBSCORE) successfully stratified individuals into risk groups based on incidence over 10 years: for example, 5.7%, 1.8%, 0.9%, 0.4% and 0.1% for cardiovascular mortality. We demonstrate generalizability of the model in independent populations of European and non-European ancestry and, in SURMOUNT-1 trial participants, show that weight loss was similar across baseline OBSCORE risk groups and that predicted risks decreased following treatment with tirzepatide. In summary, OBSCORE provides a framework for prioritizing high-risk individuals with overweight or obesity based on their risk of obesity-related complications, complementing BMI-based frameworks.

## Abstract (web summary)

OBSCORE is a machine learning-based risk prediction tool that uses a set of clinical features to stratify individuals with a body mass index (BMI) of ≥ 27 kg m^−2^ by their 10-year risk of obesity-related complications, outperforming existing models. OBSCORE is generalizable across diverse populations, supporting risk-based prioritization of obesity interventions that goes beyond simple BMI thresholds.

## Main

Obesity is a chronic disease that, if untreated, may lead to complications that range from metabolic diseases like type 2 diabetes (T2D) and cardiovascular diseases, to mechanical complications such as sleep apnea and others^1–5^. Annually, these complications account for ~5 million deaths and 130 million disability-adjusted life years, an approximately threefold increase compared to 1990^1^. Advances in obesity pharmacotherapy including semaglutide and tirzepatide targeting glucagon-like peptide-1 (GLP-1) and glucose-dependent insulinotropic polypeptide (GIP) show high efficacy in treating obesity^6–8^ and some of its complications^9–13^. As 60–70% of adults in Western countries are classified with overweight or obesity, pharmacological treatment of all individuals is a major challenge for healthcare systems^14,15^.

The identification and prioritization of individuals who are at high risk for the complications of obesity could help to guide effective allocation of resources including pharmacological, dietary, behavioral or surgical interventions but is currently hindered by important challenges. The limitations of using BMI alone as a risk measure for individuals are well recognized, and a 2025 commission report advocated for a definition of clinical obesity that considers health status and obesity subtypes beyond BMI, based on contemporary population data^16^. Yet the parameters that need to be considered to accurately identify ‘clinical obesity’ and predict its complications remain unclear, and no framework for guiding the allocation of interventions, considering differential risk, has been developed in a data-driven, risk-based manner. Large-scale, population-based studies with comprehensive baseline assessment and linkage to healthcare records now facilitate the analysis of diverse data modalities in contemporary populations^17,18^, rather than relying on historical data from selected study participants^19^. We have previously shown that a simple, interpretable machine learning framework can identify sparse sets of informative predictors among thousands of features for diverse diseases^20–22^, but this has not yet been applied to build a risk-based tool that could inform a data-driven obesity management strategy.

Here we develop and present a risk prediction tool for prioritizing high-risk individuals for interventions based on a single integrated model (OBSCORE) with 20 clinical parameters that predict the future onset of 18 important cardiovascular, metabolic and mechanical complications of obesity. We derive this model in a contemporary population of nearly 200,000 adults potentially eligible for obesity medication, by empirically integrating a comprehensive set of more than 2,300 features.

## Results

**Fig. 1:**
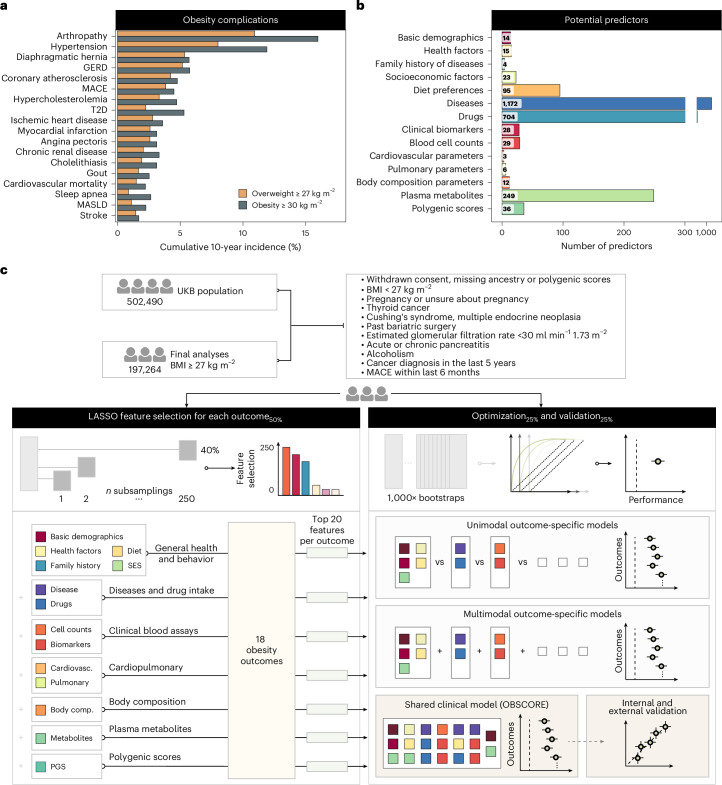
Outcomes, predictor pool and study design. **a**, Ten-year cumulative incidence of each outcome investigated in this study. **b**, Bar plot displaying the number of one-hot-encoded features considered per category. **c**, Exclusion criteria. The final dataset was split into feature selection sets (50%) and optimization (25%) and validation (25%) sets for each outcome. LASSO regression was used to select the top 20 predictive features for each set of predictors for each obesity-related outcome. We analyzed the predictive value of (1) each individual domain, (2) multimodal models, and (3) a clinically available model with a core set of shared features. The shared model was externally validated in the EPIC-Norfolk and G&H studies for available outcomes. Panel **c** created in BioRender; Blight, L. https://biorender.com/2y7grrp (2026). Cardiovasc., cardiovascular; comp., composition; MASLD, metabolic dysfunction-associated steatotic liver disease; SES, socioeconomic status. Source data

Of ~500,000 UK Biobank (UKB) participants, we included 197,264 individuals mirroring the inclusion criteria for recent weight loss medication trials^7^ (Fig. 1). We included 97,421 participants living with overweight (BMI 27–30 kg m^−^^2^) and 99,843 living with obesity (BMI ≥ 30 kg m^−^^2^), with a total of 95,034 women and 102,230 men (median age at recruitment 58 years) (Extended Data Table 1). A total of 9.3% of the included participants had T2D at baseline (13% and 5.7% in individuals with obesity and overweight (BMI ≥ 27 kg m^−^^2^), respectively) and 4.4% had a medical history of a major adverse cardiac event (MACE), including myocardial infarction or stroke. We investigated the incidence of 18 obesity-related complications as outcomes, considering the incidence of cardiovascular, metabolic and mechanical complications of obesity, as well as cardiovascular mortality over a 10-year follow-up period, excluding prevalent disease for each outcome and events occurring within the first 6 months of follow-up (Fig. 1a). Arthropathy was the most frequently occurring outcome, affecting 13.4% of the cohort, with hypertension occurring in 9.7%. A total of 9,337 deaths occurred within the follow-up period. We considered a total of 2,390 predictors from various categories, ranging from clinically available parameters such as basic demographics and clinical blood assays to more complex data such as plasma metabolite levels and polygenic scores (Fig. 1b), of which 2,078 were used as predictors after pruning for highly correlated features (*r* < 0.9). We conducted a two-step machine learning procedure to build and validate sparse models with up to 20 features and analyzed the predictive value of (1) each individual data modality (for example, general health and behavior or among others), (2) multimodal models with stepwise addition of all modalities, and (3) a clinical model with a single set of 20 shared features for prediction across all 18 outcomes (Fig. 1c). The total number of incident cases overall and in training, optimization and validation sets is depicted in Supplementary Tables 1 and 2. We externally validated the shared model in the European Investigation into Cancer (EPIC)-Norfolk^23^ and Genes & Health (G&H) studies.

### Performance of unimodal outcome-specific models

**Fig. 2:**
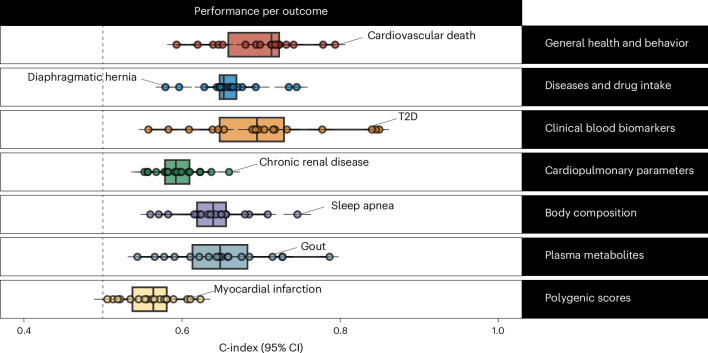
Performance of unimodal outcome-specific models. Outcome-specific models were built using the LASSO and Ridge Cox algorithms, selecting from up to 20 features from different categories of predictors individually: that is, indicators of general health and behavior, diseases and drug intake, clinical blood biomarkers, cardiopulmonary parameters, body composition, plasma metabolites and polygenic scores. Data points display the mean C-index with error bars showing 95% confidence intervals (CIs) across 1,000 bootstraps. Vertical dashed line at C-index = 0.5 denotes a random predictor. Boxplots show the median (center line) and the 25th and 75th percentiles (lower and upper box limits, respectively), and whiskers extend to the most extreme observed values within 1.5× the interquartile range from the lower and upper quartiles. Data points beyond this range are outliers. A selection of outcomes is labeled. Sample sizes are provided in Supplementary Table 2. Source data

**Extended Data Fig. 1:**
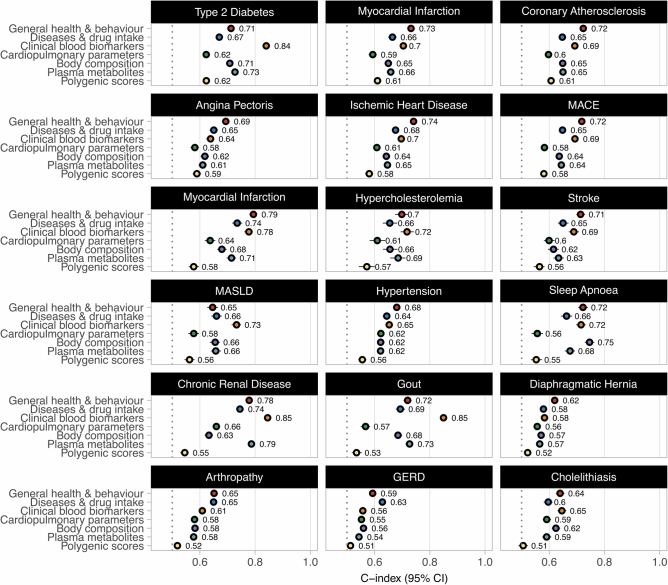
Performances of unimodal outcome-specific models. Outcome-specific models were built using the LASSO algorithm, selecting from up to 20 features from different categories of predictors individually, that is, indicators of general health and behavior, diseases and drug intake, clinical blood biomarkers, cardiopulmonary parameters, body composition, plasma metabolites, and polygenic scores. The Concordance-index (C-index) with 95% confidence intervals computed using 1,000 bootstrap resamples is plotted for each outcome. Vertical dashed line at C-index = 0.5 denotes a random predictor. Supplementary Table 2 contains sample size information. Source data

We compared the discriminative performance of models including the top 20 features from each data modality (that is, feature set) individually (Fig. 2, Extended Data Fig. 1 and Supplementary Table 3). We observed a wide range of performance by individual domains for the prediction of the complications of obesity, with general health and behavior (median concordance index (C-index) 0.713, range 0.593–0.794) and clinical blood biomarkers (median C-index 0.695, range 0.557–0.850) achieving the best performance and polygenic scores achieving the lowest performance (median C-index 0.564; range 0.506–0.623). Sensitivity analyses including all non-random features of individual domains in the models instead of only the 20 top features demonstrated highly similar performance (Supplementary Fig. 1). We observed no apparent increase in C-indices for most domains apart from increases for the diseases and medication domain (median delta C-index 0.02, range 0.005–0.033).

### Performance of multimodal outcome-specific models

**Fig. 3:**
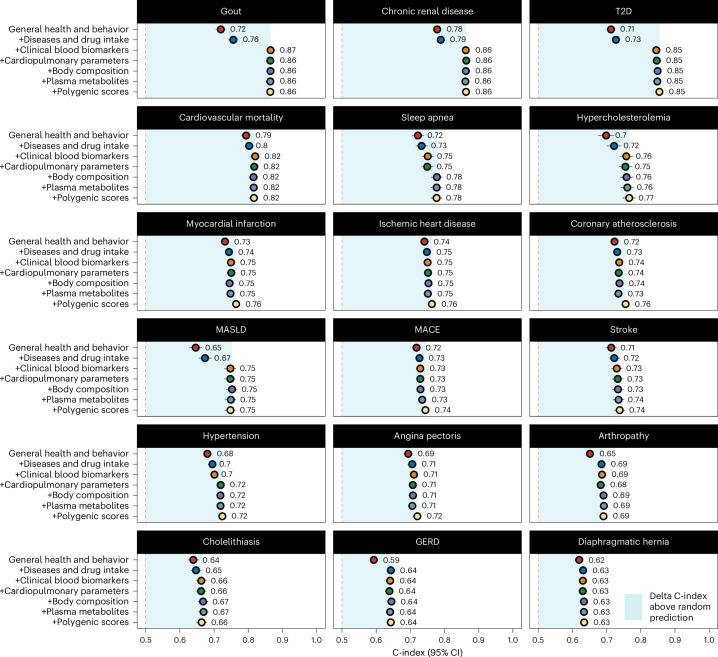
Performance of multimodal outcome-specific models. Outcome-specific models were built using LASSO and Ridge Cox models, selecting the top 20 features from increasingly comprehensive pools of predictors, for each outcome separately. The predictors were added stepwise in the following order: (1) indicators of general health and behavior, (2) diseases and drug intake, (3) clinical blood biomarkers, (4) cardiopulmonary parameters, (5) body composition, (6) plasma metabolites and (7) polygenic scores. Data points display the mean C-index with error bars showing 95% CIs across 1,000 bootstrap tests. Vertical dotted line at C-index = 0.5 denotes a random predictor. Blue shaded area depicts the maximum increase in C-index over random prediction. Sample sizes are provided in Supplementary Table 2. Source data

We next analyzed discriminative performance when adding different data domains, by selecting the top 20 features from increasingly comprehensive pools of predictors for each outcome separately in the following order: (1) indicators of general health and behavior, (2) diseases and drug intake, (3) clinical blood biomarkers, (4) cardiopulmonary parameters, (5) body composition, (6) plasma metabolites and (7) polygenic scores (Fig. 3 and Supplementary Table 4).

**Extended Data Fig. 2:**
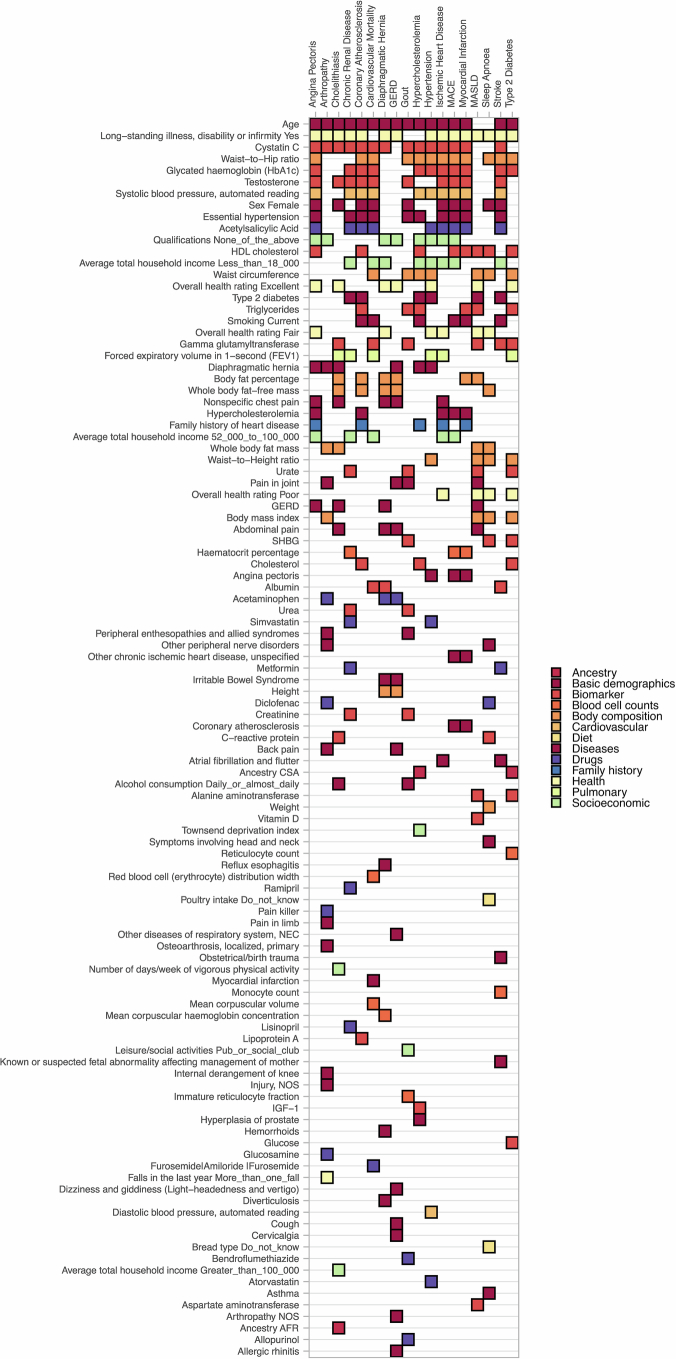
Features from clinical models. Top 20 features selected in each multimodal outcome-specific model are depicted, filled by category of the feature. The pool of features includes general health & behaviour, diseases and medication, clinical blood biomarkers, cardiopulmonary parameters and body composition. Source data

**Extended Data Fig. 3:**
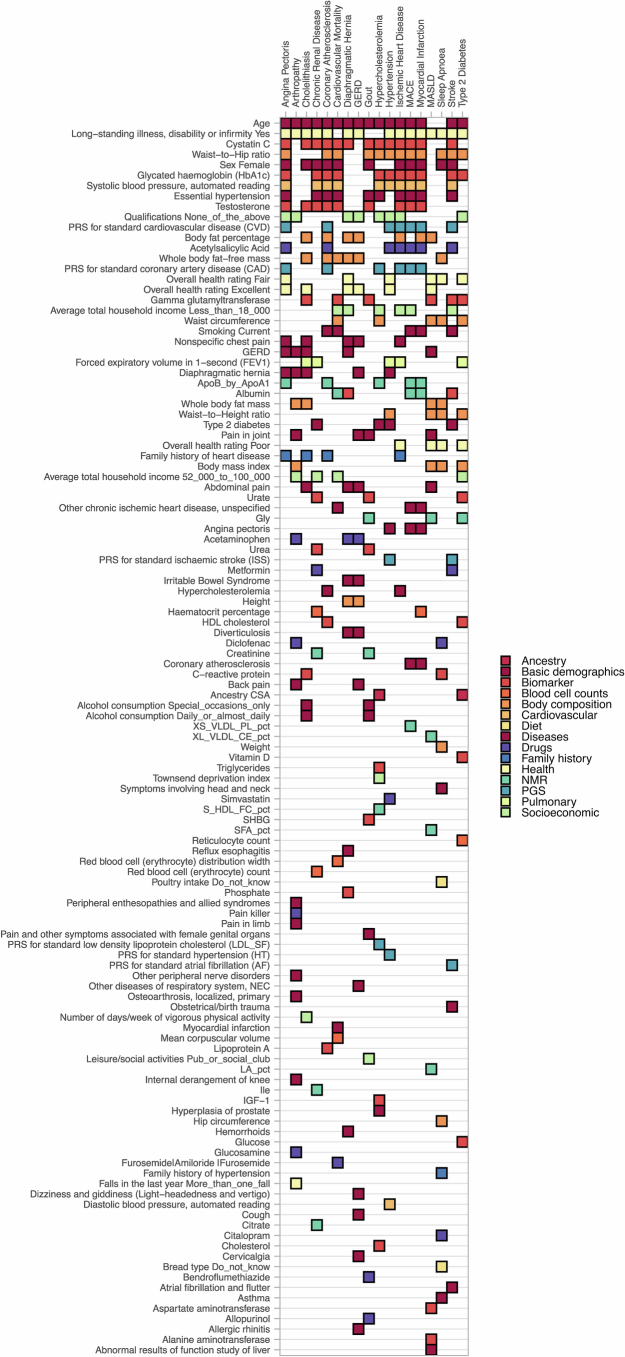
Features from extended models. Top 20 features selected in each multimodal outcome-specific model are depicted, filled by category of the feature. The pool of features includes general health & behavior, diseases and medication, clinical blood biomarkers, cardiopulmonary parameters and body composition, plasma metabolites, and polygenic scores. Source data

For most outcomes, the integration of current and past diagnoses, current medication and clinical blood assays on top of general health and behavior parameters led to increases in C-indices (increase in median mean C-index 0.025). This emphasizes the large predictive value of information mostly available in clinical records. However, further increasing the predictor pool to select from cardiopulmonary parameters (change in median mean C-index −0.0006), body composition (increase in median mean C-index 0.002), plasma metabolites (change in median mean C-index −0.0003 or polygenic scores (increase in median mean C-index 0.003) provided limited additional gains in predictive accuracy for most outcomes. Notably, although body composition markers are well-known predictors of the complications of obesity, the addition of these measures resulted in relatively small increases in performance across all outcomes. The largest increase in performance with the addition of body composition was observed for sleep apnea (0.027). Augmenting the potential predictor pool to all modalities of data, predictive performance varied substantially across outcomes (median C-index 0.75, range 0.634–0.864). The highest performance was observed for gout (0.864 (95% CI 0.852–0.876)), chronic kidney disease (0.863 (95% CI 0.852–0.872)) and T2D (0.854 (95% CI 0.846–0.863)), largely driven by clinical blood assays. By contrast, the lowest performance was observed for diaphragmatic hernia and gastroesophageal reflux disease (GERD), with maximum C-indices of 0.634 (95% CI 0.623–0.646) and 0.643 (95% CI 0.63–0.654), respectively. Outcome-specific features most frequently selected across outcomes are displayed in Extended Data Fig. 2 for clinically available models (excluding plasma metabolites and polygenic scores) and Extended Data Fig. 3 for extended models.

### Performance of the data-driven shared clinical model OBSCORE

**Fig. 4:**
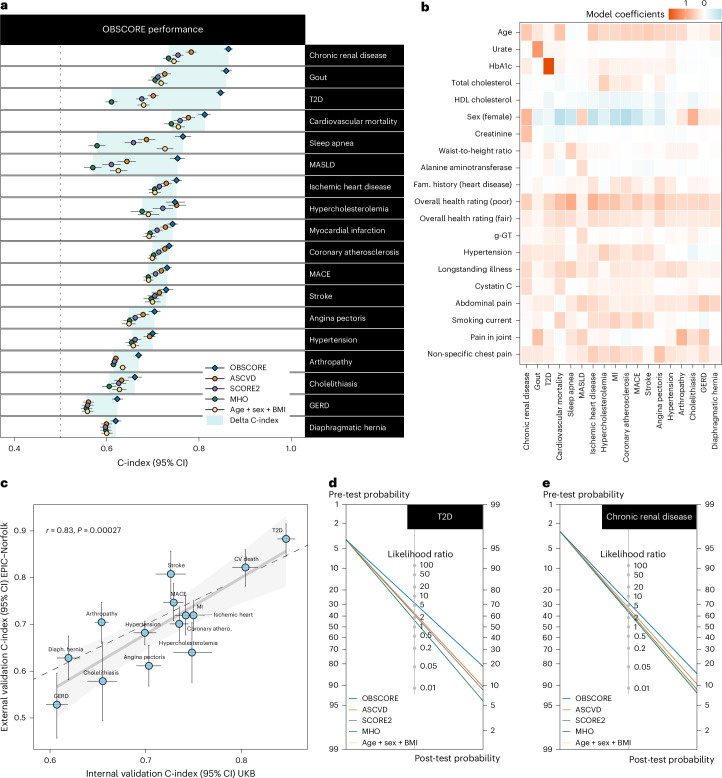
Features and performance of OBSCORE. **a**, Performance of OBSCORE in comparison with age, sex and BMI, as well as features from other risk models, such as MHO, SCORE2 risk score and ASCVD risk estimator. Data points display the mean C-index, with error bars showing 95% CIs estimated from 1,000 bootstrap resamples. Sample sizes are provided in Supplementary Table 2. **b**, Top 20 features identified and used in OBSCORE and their coefficients across all outcomes. **c**, External validation of OBSCORE (with 18 features) performance in the EPIC-Norfolk study for 14 outcomes. Data points display the mean C-index with 95% CIs estimated from 1,000 bootstrap samples. The solid line represents the fitted linear regression model, with shaded bands indicating 95% CIs. Two-sided Pearson’s correlation test was used to derive the raw *P* value and the correlation coefficient (*r*). **d**, Fagan’s nomogram for T2D displaying pretest and post-test probabilities using different models. **e**, Fagan’s nomogram for chronic renal disease displaying pretest and post-test probabilities using different models. Pretest probability is calculated based on incidence in the study, and post-test probability is calculated based on likelihood ratio for each model at 10% FPR. Athero., atherosclerosis; diaph., diaphragmatic; fam., family. Source data

**Extended Data Fig. 4:**
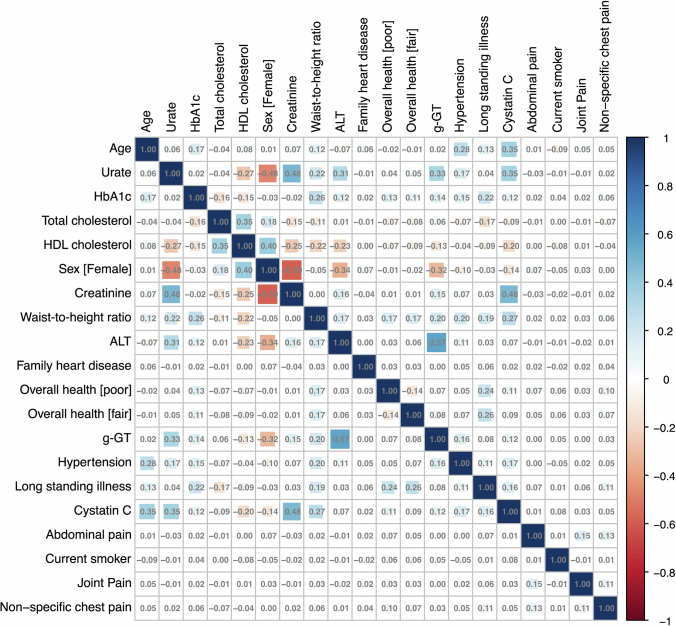
Correlation matrix of features in OBSCORE. Two-sided Pearsons’ correlation test was used to compute correlation coefficients. Source data

Implementing separate predictive models for each health outcome is impractical in clinical settings and would not result in an integrated assessment of overall risk. We investigated shared predictors among top features based on the training set for the 18 outcome-specific models, with the goal of identifying common predictors that could inform a unified model applicable to multiple outcomes^20,24^. We prioritized rapidly available clinical features over more sophisticated markers such as serum metabolites and polygenic scores, owing to only small prognostic value, and identified a shared model (OBSCORE) with a set of 20 largely uncorrelated features (Fig. 4a,b, Extended Data Fig. 4 and Supplementary Table 5).

**Extended Data Fig. 5:**
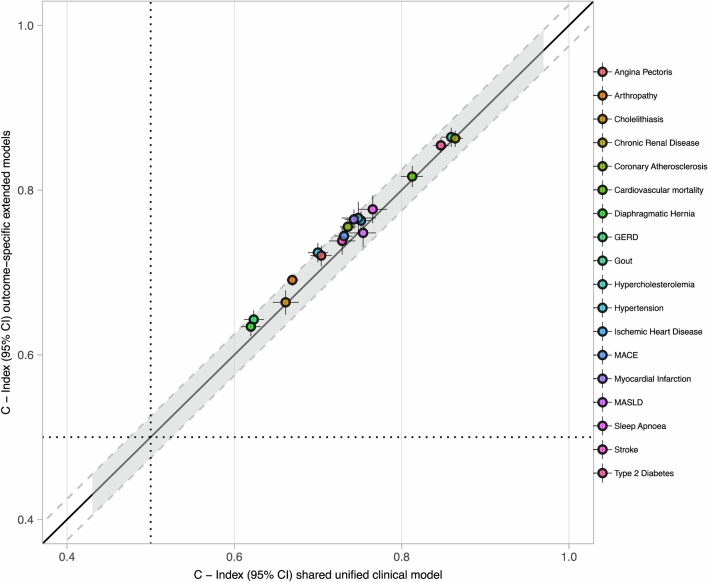
OBSCORE versus extended outcome-specific models. The performances (expressed as Concordance index with 95% confidence intervals computed using 1000 bootstrap resamples) of the shared model (X-axis) are compared with performances of extended outcome-specific models (Y-axis). Dashed lines represent 2.5% deviation intervals. Dotted lines represent a random predictor (C-index = 0.5). Source data

In the validation sets, OBSCORE achieved discriminative performance comparable with the extended outcome-specific models (that is, those including top-20 features from all data modalities), with an overall median deviation in C-index across outcomes of 0.012 (range −0.006–0.024) (Extended Data Fig. 5).

**Extended Data Fig. 6:**
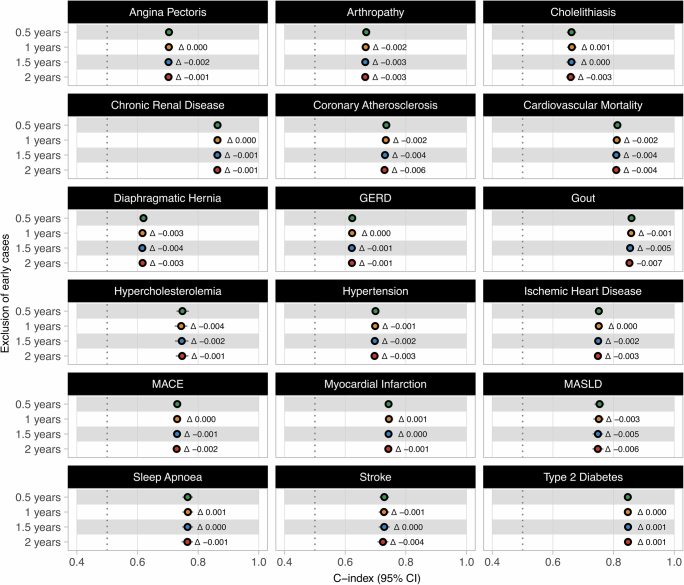
Sensitivity analyses with extended exclusion of early incident cases. In addition to the main analyses excluding incident cases occurring in the first 6 months, performances (expressed as Concordance index (C-index) with 95% confidence intervals estimated using 1,000 bootstrap resamples) of OBSCORE were tested when excluding cases occurring within 1, 1.5, and 2 years after baseline. Source data

**Extended Data Fig. 7:**
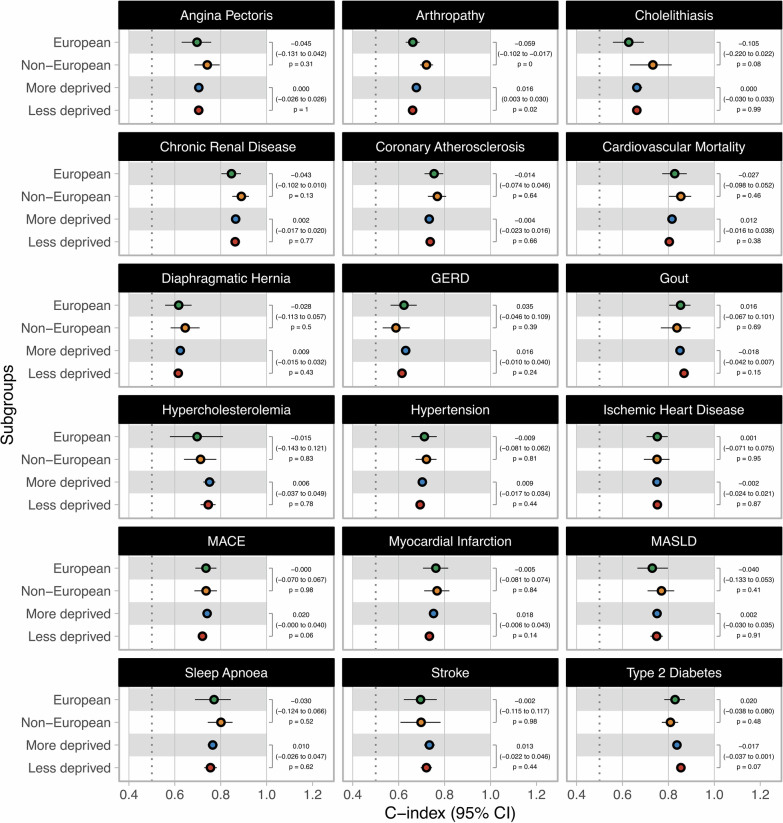
Subgroup analyses in the UK Biobank. Performances of OBSCORE in individuals of European and non-European ancestry and lower and higher deprivation subgroups (cut-off cohort median of Townsend deprivation index, corresponding to −2.05). Highlighted are mean C-indices and differences between subgroups with differences 95% confidence intervals and p-values estimated from comparing 1000 bootstrap samples. Source data

We next compared OBSCORE to a model consisting of age, sex and BMI, as well as the well-recognized definition of metabolically healthy obesity (MHO). Considering the unavailability of similar obesity-complication focused scores, we compared the performance of OBSCORE with features from established risk predictors such as SCORE2 and atherosclerotic cardiovascular disease (ASCVD) risk estimator (Fig. 4b and Supplementary Table 6). OBSCORE outperformed the BMI-based approach across all outcomes (Fig. 4b). Across 17 outcomes, OBSCORE outperformed ASCVD features (median difference in C-index of 0.027, range −0.004–0.146). Although ASCVD features performed comparably to OBSCORE for some cardiovascular outcomes, such as hypertension and coronary atherosclerosis, we observed a considerable difference for death from cardiovascular causes (difference in mean C-index 0.035). Particularly for non-cardiovascular outcomes, there were stark differences: for example, for T2D (mean C-index difference = 0.146) and sleep apnea (C-index difference = 0.079). Performance did not differ when excluding early incident cases up to 2 years after baseline in a sensitivity analysis (Extended Data Fig. 6). In subgroup analyses, model performance was similar comparing European to non-European ancestries or groups of lower versus higher Townsend deprivation index (Extended Data Fig. 7). We tested the generalizability and performance of OBSCORE through external validation in the prospective EPIC-Norfolk cohort study. We used coefficients derived in UKB—that is, without model retraining—for 18 of the 20 features available in EPIC-Norfolk to predict 14 outcomes (range of case numbers = 48–217). We observed strong agreement in performance (*r* = 0.83; *P* = 2.7 × 10^−4^), indicating high generalizability of the model (Fig. 4c). We further tested the generalizability of our model to individuals of South Asian ancestry from the G&H study, a population at high risk of developing T2D early and at lower levels of BMI compared to white Europeans. Here, 15 available OBSCORE features predicted incident T2D well (mean C-index = 0.78) and achieved a greater improvement (difference in mean C-index) of 0.196, compared to 0.166 in UKB over the basic model including age, sex and BMI. This indicates generalizability of the features selected, even in populations with different profiles and study methods.

Beyond C-indices, screening metrics including detection rates across different false positive rate (FPR) thresholds ranging from 5% to 40% were similar between the shared clinical and outcome-specific extended models (Supplementary Fig. 2). The probability of a high-risk individual, identified through OBSCORE, developing obesity-associated complications (that is, post-test probabilities) at a 10% FPR ranged from 4.8% to 28.7% (median = 10%) across the 18 outcomes studied, compared to 3.9–24.5% (median = 8.5%) for high-risk individuals identified by ASCVD features. For example, post-test probabilities at 10% FPR for OBSCORE were as high as 18.5% for T2D, an approximately twofold increase compared to post-test probabilities from other models (Fig. 4d,e). Pretest and post-test probabilities at 10% FPR for the other outcomes are displayed in Supplementary Figs. 3 and 4. We evaluated calibration in the held-out validation sets. Most outcomes were well calibrated, although OBSCORE overestimated risk for some outcomes (Supplementary Fig. 5). Applying Platt’s logistic regression improved calibration across outcomes.

### Risk stratification by OBSCORE

**Fig. 5:**
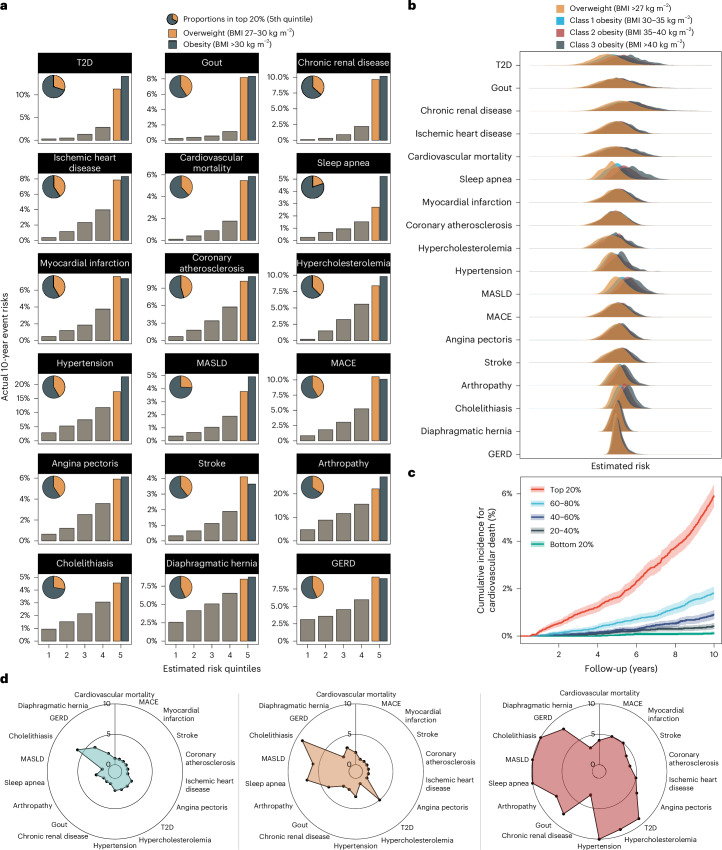
Risk stratification by OBSCORE. **a**, Ten-year observed risk rates across outcomes, according to groups (each containing 20% of individuals) of risk states as estimated by OBSCORE. The bar of the highest 20% (5th quintile) shows event rates stratified by individuals with overweight (BMI 27–30 kg m^−^^2^) and obesity BMI >30 kg m^−^^2^. Embedded pie charts depict the proportions of individuals with overweight versus obesity in the highest 20%. **b**, Distribution of risk estimates in individuals stratified by overweight and class 1, class 2 and class 3 obesities. **c**, Cumulative incidence plot for death from cardiovascular causes according to the bottom 20%, 20–40%, 40–60%, 60–80% and top 20% of estimated risk. 1 − Kaplan–Meier estimates are shown, and error bands represent 95% CIs. **d**, Radar plots depict the predicted risks of three individuals with the same age, sex and BMI for the 10-year development of 18 obesity-associated complications. Risk deciles are 10% risk groups as compared with the underlying reference group in the UKB. Source data

**Extended Data Fig. 8:**
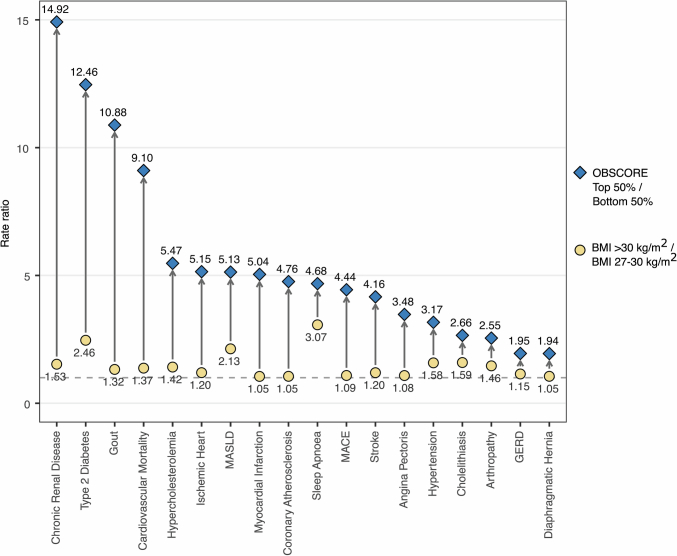
Risk stratification comparing OBSCORE and obesity/overweight classification. Ratios of number of observed events in individuals with 50% highest predicted OBSCORE vs. lowest 50% predicted OBSCORE risks and ratios of number of observed events in individuals living with obesity vs individuals with overweight, across outcomes. Source data

The utility of predictive models lies in their ability to stratify individuals by absolute risk, enabling targeted interventions and informed clinical decision-making. To evaluate this stratification ability, we analyzed the observed numbers of events across 10 years for estimated risk groups, as depicted in Fig. 5a. We observed large proportions of participants with overweight instead of obesity, even in the highest fifth of risk scores (Fig. 5a), highlighting limitations of BMI-only–based approaches to prioritize obesity complication risk. Across outcomes, the proportion of individuals with BMI 27–30 kg m^−^^2^ in the highest risk decile ranged from 19% to 45% (median = 39.9%). Notably, despite being among the most proximal outcomes of obesity, the proportion of BMI 27–30 kg m^−^^2^ among individuals at highest risk for T2D was 30%. To further investigate the large differences in risk despite similar BMI, we investigated the distributions of estimated risk stratified by overweight BMI 27–30 kg m^−^^2^, class 1, class 2 and class 3 obesities (Fig. 5b). Within each stratum, we observed wide-ranging risk differences for most outcomes, despite equal BMI category. This overlap was smaller for outcomes more proximally related to body weight, such as metabolic dysfunction-associated steatotic liver disease. OBSCORE stratified risk across all outcomes (that is, rate ratios were consistently more than 1 between the top and bottom 20%). For 12 out of 18 outcomes, OBSCORE demonstrated substantial stratification (rate ratio > 10). Rate ratios were as high as 89-fold for chronic renal disease, 36 for gout and 42 for T2D. Notably, the rate ratio for death by cardiovascular cause was 47, with a 10-year absolute event rate of 5.7% in 20% of the individuals with the highest risk. Cumulative 10-year incidences for cardiovascular mortality across all quintiles are shown in Fig. 5c. We note that the rate ratios of this magnitude warrant particular attention, as we did not compare to participants within the reference BMI range, but all participants, even in the lowest 20%, were living with overweight (BMI 27–30 kg m^−^^2^) or obesity. Moreover, rate ratios comparing individuals in the top to bottom 50% of OBSCORE were higher (range = 1.9–14.9) than those comparing individuals with a BMI of >30 kg m^−^^2^ to those with a BMI of 27–30 kg m^−^^2^ (range = 1.05–3.07) (Extended Data Fig. 8). We demonstrate clinical heterogeneity by exemplary illustrating the risk of developing 18 outcomes for three selected individuals with the same age, sex and BMI (Fig. 5d). We developed an interactive web interface (https://omicscience.org/apps/obscore/) to enable interrogation and exploration of model-derived individual-level risk estimates.

### OBSCORE in a randomized controlled trial of tirzepatide

**Fig. 6:**
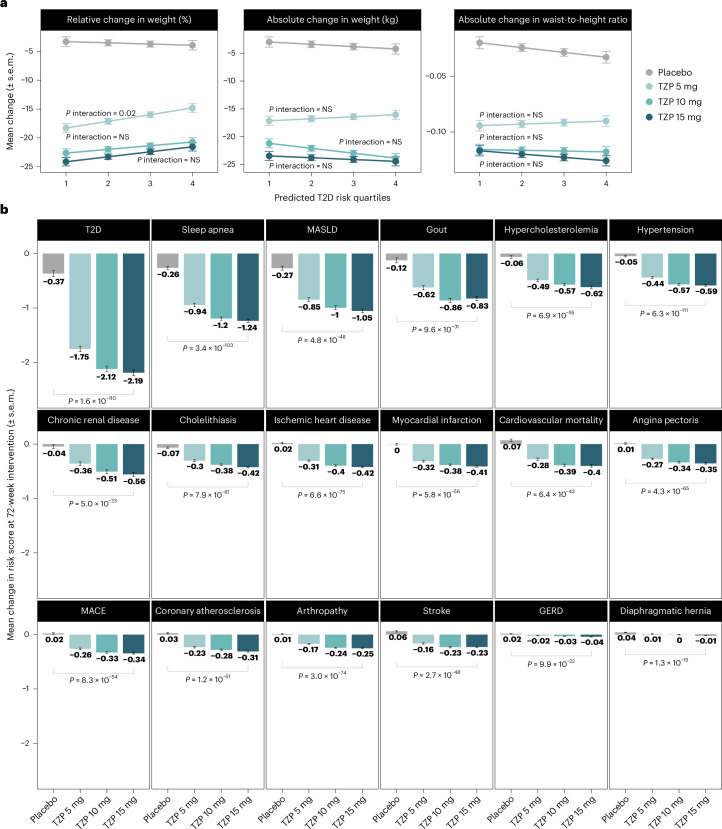
OBSCORE in the SURMOUNT-1 trial. **a**, EMMs of change in percent body weight, body weight and waist-to-height ratio along with ±s.e.m. at week 72 after treatment based on predicted T2D risk quartiles in the SURMOUNT-1 trial. Unadjusted interaction *P* values (treatment × risk quartiles) were obtained from linear regression. *n*_placebo_ = (Q1 = 89, Q2 = 103, Q3 = 101, Q4 = 95), *n*_TZP 5mg_ = (Q1 = 116, Q2 = 104, Q3 = 126, Q4 = 116), *n*_TZP 10mg_ = (Q1 = 121, Q2 = 127, Q3 = 112, Q4 = 119), *n*_TZP 15mg_ = (Q1 = 125, Q2 = 117, Q3 = 112, Q4 = 121). **b**, EMMs of change in predicted risk scores (linear predictors) across 18 outcomes at week 72 (±s.e.m.) in placebo and treatment arms. *n*_placebo_ = 388, *n*_TZP 5mg_ = 462, *n*_TZP 10mg_ = 479, *n*_TZP 15mg_ = 475. Unadjusted *P* values for two-sided pairwise comparisons between 15 mg Ttrzepatide (TZP) and placebo are shown. NS, not significant. Source data

**Extended Data Fig. 9:**
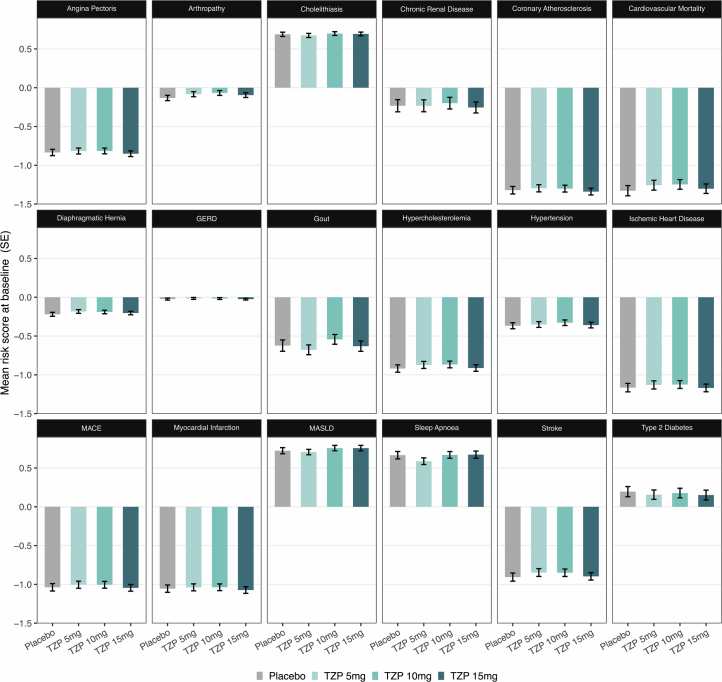
Baseline OBSCORE (12 features) risk scores across treatment arms and outcomes in the SURMOUNT-1 trial. Mean risk scores at baseline with standard errors of the mean are shown. n_placebo_ = 388, n_TZP 5mg_ = 462, n_TZP 10mg_ = 479, n_TZP 15mg_ = 475. Source data

To test whether changes in adiposity measures by tirzepatide treatment were similar across OBSCORE risk groups and whether OBSCORE captured treatment-induced changes in risk, we applied our model with 12 of the 20 features available to individual-level data from the SURMOUNT-1 trial^7^. We observed comparable reductions in body weight and waist-to-height ratio with tirzepatide (72 weeks) across OBSCORE-predicted risk groups at baseline, with only one of 162 comparisons reaching the multiple-testing-corrected significance threshold (Supplementary Table 7). Absolute changes in weight and waist-to-height ratio were on average greater in individuals with higher predicted OBSCORE risk, whereas relative changes, such as in weight loss, were slightly smaller—for example, for T2D (Fig. 6a)—consistent with findings in SURMOUNT-2^25^. At baseline, OBSCORE-predicted risks in the treatment versus placebo arms did not differ (Extended Data Fig. 9), but they were significantly lower in all tirzepatide arms after the 72-week follow-up across all outcomes (Fig. 6b and Supplementary Table 8), indicating that OBSCORE captured treatment-associated changes in predicted risk.

## Discussion

In this study of ~200,000 individuals with overweight (BMI 27–30 kg m^−^^2^) or obesity, we systematically investigated the performance of different data modalities, individually or in combination, for predicting the 10-year incidence of 18 obesity-associated complications. Our agnostic framework identified a shared predictive model (OBSCORE) that can effectively predict these complications and may inform risk-based allocation of obesity medication as well as other dietary, behavioral or surgical interventions.

New obesity medications targeting hunger signaling via GLP-1 and in combination with GIP and glucagon represent a major advance in obesity management. Comparing with bariatric surgery, recent data indicate that short-term weight management and outcomes for complications may be similar^26^. Hence, these pharmacological interventions pose a promising option to manage overweight and obesity at population scale, globally^27^. The ultimate goal of these interventions is to prevent obesity-associated outcomes. However, there is currently no consensus for identifying individuals at highest risk for obesity-associated complications, who could be prioritized for allocation of the new medications. Besides clinicians and clinical routine, this uncertainty also affects other key stakeholders, such as policymakers, healthcare systems and insurance providers, with implications for how access is determined^28^. A framework for prioritization is important for several reasons, including the current high costs^28,29^, global shortages^30^, lack of data-driven support tools for clinicians and targeting of drugs to those at highest absolute risk. Currently, recommendations for the allocation of these therapies remain underdeveloped. For instance, owing to scarcity of data and data-driven approaches providing a rationale for prioritization, the US health insurance program Medicare limited the coverage of semaglutide to a specific set of individuals with obesity and known history of heart disease or stroke^31,32^. OBSCORE, however, identified many individuals with overweight (BMI 27–30 kg m^−^^2^) with other risk factors at similarly high risk for complications. For individuals identified as high-risk despite having a BMI less than 27 kg m^−^^2^, it remains to be determined whether targeted interventions provide clinical benefit, as current trials mostly focused on individuals with overweight or obesity. Whether different cut-offs for BMI should be considered is particularly important for certain populations who have a higher risk of obesity-related complications at lower BMI cut-offs. Moreover, such criteria often neglect non-cardiovascular outcomes, although for some of them, benefit has been demonstrated^27^. Further, challenging the commonly practiced BMI-based prioritization, our results highlight how large intraindividual discordance for cardiovascular/non-cardiovascular complications can be, even among individuals with a similar BMI. Overall, our data-driven approach aims to suggest an agnostically derived set of predictors that may be considered for identifying individuals at high risk for a broad set of obesity complications.

BMI thresholds are the currently implemented, simple mechanism for establishing eligibility for behavioral and pharmacological obesity management intervention. Recent consort statements^16^ have highlighted the need to develop frameworks that better align eligibility with adiposity-associated clinical risks. Our findings suggest that OBSCORE could be useful for refining existing BMI-based criteria by identifying individuals with overweight and obesity and with a risk profile comparable to or greater than individuals currently eligible based on BMI cut-offs. Ideally, future studies will further validate and calibrate the model in additional populations and with sensitive and specific follow-up for diverse obesity-related outcomes. We envisage OBSCORE to be implemented as a data-driven support tool for referral that complements existing frameworks, rather than requiring a drastic change of regulatory indications or reimbursement policies. Within systems such as NICE in the UK, OBSCORE could be incorporated at the level of technology appraisal and clinical guidelines. To operationalize targeting of ‘high-risk’ OBSCORE individuals, processes to ensure the availability and clinical documentation of parameters required for standardized, quantitative risk estimation would need to be implemented. The fact that OBSCORE is based on a minimal set of 20 features, most of which are readily available in a clinical setting, may facilitate implementation in electronic health record (EHR)-based decision support, for (semi)automated identification of eligible patients at the point of care.

This approach is a complementary perspective to concepts such as ‘obesities’^33^ and MHO^34,35^ or ‘discordant obesity’^36,37^. Each of these concepts aims to identify and explain facets of heterogeneity in obesity, and they have advanced our understanding and clinical view of obesity. However, these approaches so far have considered a set of prespecified cross-sectional features to define heterogeneity. By contrast, our approach has empirically derived most informative features, based on their longitudinal associations with outcomes, to identify clinical obesity, as recently highlighted^16^. Nevertheless, future work should determine whether OBSCORE’s sparse combination of features could be further refined in the context of cost-effectiveness and patient outcomes.

Using individual-level data from the SURMOUNT-1 trial, we demonstrated that tirzepatide-related changes in weight and waist-to-height ratio were evident and comparable across OBSCORE risk groups at baseline, suggesting that treatment efficacy demonstrated in the trial^7^ may be generalizable to individuals prioritized by our model. In combination with their higher predicted risk of important obesity-related outcomes, this further highlights the potential opportunity for targeted intervention in this group.

Some limitations should be acknowledged when interpreting our results. Our study included individuals of middle to older ages. Considering the dramatic rise of obesity among adolescents and younger adults^14^, future research must evaluate the generalizability of our model to those age groups and other populations not represented in this study. However, we observed good performance across non-European ancestries and across Townsend deprivation groups. The UKB represents a healthier population compared to the general population, leading to an underestimation of event rates^38^. Although this impacts absolute risk estimates, it is unlikely to substantially affect the model-building process or model performance. Hence, although discriminative performance was strong and can stratify the population into statistically defined high and low risk individuals, ultimate reliability on absolute risks for clinical decision-making will require future research on (1) informed absolute risk cut-offs for several of these diseases yet needed to be established by the clinical community and (2) calibration with larger-scale and diverse cohorts or trials with stricter case ascertainment. Leveraging EHR data to ascertain outcomes may have introduced potential biases^39^. Although the predictors identified in our analysis are clinically plausible and partially consistent with other models, the study design was not intended to establish causal predictors, and hence these should be interpreted with caution. Some outcomes with high incidence rates, such as arthropathy and GERD, displayed lower C-indices, necessitating further studies with other data modalities to establish stronger predictors.

The comprehensive and unbiased approach marks an important strength of our study. We leveraged a large and diverse set of more than 2,300 predictors in ~200,000 individuals with overweight and obesity. Our approach represents one of the largest studies of individuals with overweight or obesity with data on 249 plasma metabolites. Moreover, we covered a range of domains including easily accessible information on general health and behavior, a deep characterization of medical history with more than 1,000 current diagnoses and more than 700 different medications, most of the clinically relevant blood biomarkers and blood cell counts, clinically accessible cardiopulmonary measurements, different body composition parameters and established polygenic scores for 36 different diseases or traits. Our use of an interpretable machine learning framework refined through our prior work^20–22^ enabled us to distill this large set into a sparse subset. These predictors were selected in a data-driven manner and with minimal manual curation, and are both clinically plausible and implementable in real-world settings. We were able to externally validate OBSCORE for 14 out of 18 outcomes tested in independent studies, showing overall generalizability of OBSCORE and retained performance of the models, although remaining outcomes need further testing. Notably, external validation showed robust results even though there were differences in methods between the studies: for example, despite EPIC-Norfolk participants being recruited ~15–20 years earlier. This consistency emphasizes the potential for real-world applications.

In conclusion, we developed and validated a risk prediction tool to identify individuals at high risk of developing obesity-related complications. These findings may inform data-driven, risk-centered tailoring of obesity management strategies.

## Methods

All studies were conducted according to all relevant ethical regulations and principles of the Declaration of Helsinki. UKB was granted ethical approval from the North West Centre for Research Ethics Committee (11/NW/0382), in the form of a Research Tissue Bank (RTB) approval. The EPIC-Norfolk study has been approved by the Norfolk Research Ethics Committee, under the reference number 05/Q0101/191. G&H was approved by the London Southeast NRES Committee of the Health Research Authority (14/LO/1240). SURMOUNT-1 was conducted according to the principles of the Declaration of Helsinki and Good Clinical Practice guidelines and was approved by independent ethics committees or institutional review boards at each of the 119 sites across nine countries^7^.

### Statistics and reproducibility

The UKB is a prospective, population-based observational study of ~500,000 individuals residing in the UK, aged 40–69 at enrollment, who were recruited between 2006 and 2010 across >20 centers^17^. All participants provided informed consent, and study procedures were performed according to the Helsinki declaration. This study was conducted with data accessed under the application numbers 44448 and 30418.

No statistical method was used to predetermine sample size. No randomization or blinding was applied in this observational study. To increase generalizability and reflect a scenario close to clinical reality, we applied exclusion criteria closely matching those of the SURMOUNT-1 trial^7^ to define the study population (Fig. 1c). However, we did not exclude individuals with prevalent T2D because this population has been investigated separately in SURMOUNT-2^25^, and we did not exclude individuals with weight change >5 kg within the last 90 days because this information was not reliably obtainable^7^. We included participants with a BMI equal or above 27 kg m^−2^ with or without prevalent comorbidities, and we excluded participants with contraindications for dual agonist therapies, with self-reported pregnancy or uncertainty about pregnancy or with diagnoses of thyroid cancer^7^. Additionally, we excluded participants with other potential causes of obesity: that is, owing to Cushing’s syndrome or reported intake of tricyclic antidepressants, atypical antipsychotics, mood stabilizers or paroxetine. We excluded participants with a history of bariatric surgery, estimated glomerular filtration rate < 30 ml min^−1^ per 1.73 m^2^, acute or chronic pancreatitis, alcoholism, recent cancer diagnosis (within the last 5 years) or recent MACE within the last 6 months. After applying these criteria, 197,264 participants remained for the final analyses. Analyses are based on distinct samples and did not include technical or biological replicates. All tests were two-sided.

### Exposure

To establish a data-driven discovery of predictors of obesity-associated complications, we collated a comprehensive dataset of 2,390 exposure variables by leveraging multimodal data^40^ (Supplementary Table 9).

We aggregated data on general characteristics of participants, including data on age, sex (self-reported), self-reported overall health, socioeconomic status, deprivation indices and related factors. We included behavior parameters, such as dietary habits and activity levels. To integrate medical history of the participants, we have gathered a comprehensive set of prevalent medical conditions for each participant from different data sources including self-reported conditions, primary care records where available (~45% of the study population), hospital episode statistics and cancer registries, as outlined previously^41^. We mapped these medical conditions onto ‘phecodes’—that is, medical concept terms—as described^41^. We use the term disease/diagnosis rather ‘phecode’ for accessibility.

We included information on regular medication and health supplements from self-report, which were categorized based on the Anatomical Therapeutic Chemical classification system as described previously^41^, yielding a total of 704 individual Anatomical Therapeutic Chemical codes. We have included a wide-ranging set of clinically established blood biomarkers and blood cell counts, the measurement of which have been described before^42^. We collated basic cardiopulmonary measurements including heart rate, blood pressure and spirometry parameters. In addition to basic body composition parameters (for example, weight, height, BMI, waist circumference, hip circumference and waist-to-hip and waist-to-height ratios), we considered bioelectrical impedance measures. We included metabolites measured with the Nightingale platform using the nuclear magnetic resonance method^43,44^. We included polygenic scores for a diverse set of 36 different diseases and traits, as provided by the UKB^45^.

After filtering highly correlated variables (*r* > 0.9), whereby variables were kept either due to lower missingness or clinical availability, a total of 2,078 features were included in the predictor pool. We applied one-hot encoding to categorical variables, transforming them into binary indicator variables. We standardized continuous variables using *Z*-score transformation, where each value was centered by subtracting the variable mean and scaled by dividing by its standard deviation. We used multiple imputation to impute missing data using the miceRanger package (version 1.5.0), excluding variables with missingness above 25%, and generated a single dataset with five iterations^46^.

### Outcomes

We computed prediction models for 18 different obesity outcomes^3–5^. We set the recruitment date in UKB as the baseline of the observation period. Follow-up was censored at 10 years based on the time of event for the outcome or date of death as retrieved from the death register, whichever occurred first. Incident complications were defined based on phecode definitions as described in detail previously^41^, from primary care where available, hospital episode statistics and self-report, except for T2D, for which we adapted a previously described definition to correctly distinguish between the different types, and for myocardial infarction and stroke, for which we adapted algorithmically defined outcomes provided by UKB^47^ (Supplementary Table 10). For each outcome investigated, we removed prevalent cases (additionally using tests that were available at baseline, such as blood pressure measurements for hypertension). To minimize bias arising from delayed diagnosis, we excluded incident complications occurring within the first 6 months of follow-up. The number of cases based on availability of primary care data are shown in Supplementary Table 11.

### Statistical analysis

#### Feature selection, optimization and internal validation

We employed a two-step machine learning procedure to identify sets of predictors consisting of the 20 most important features for each of the 18 complications, as recently applied to identify proteomic signatures predicting diseases^20,21^. This procedure involves first applying a least absolute shrinkage and selection operator (LASSO) model to identify the most relevant predictors from a large pool of features. In the second step, these selected predictors are used in a regularized Cox proportional hazards model for optimization and validation, providing a robust framework for feature selection and outcome prediction.

We randomly divided the cohort of eligible participants into three sets for each outcome: (1) feature selection (50%), (2) optimization (25%), and (3) validation (25%). In the feature selection set for each outcome, we applied LASSO with 250 random subsamples, each containing a fraction of 40% of the feature selection set. For each outcome, we compared different pools of possibly selectable features: that is, either by comparing each category (for example blood biomarkers, diseases and drugs and plasma metabolites, among others) of features individually or by increasing the pool of features by adding categories in a stepwise manner. For each outcome and from each set of feature pools, we took forward features with the top 20 highest feature selection scores—that is, those selected most frequently across subsamples—to be used in the optimization and validation sets. Our approach, refined over iterations for similar feature selection use cases^20–22^, is conceptionally equivalent to stability selection strategies^48^. In the optimization and test sets, we used a Cox proportional hazards model with *L*_2_ regularization (ridge). We performed fivefold cross-validation on the optimization set to identify the optimal lambda parameter using a fine grid. For validation, we implemented a bootstrap procedure with 1,000 bootstrap samples to assess model performance on the independent test dataset. In each bootstrap sample, we calculated the C-index to serve as a discriminatory index for predictive performance. We took forward the average C-index and 95% CIs using the 2.5th and 97.5th percentiles of the bootstrap distribution.

#### Derivation and validation of a shared model (OBSCORE) for all outcomes

In a clinical setting, implementation of a single model with a core set of features that predict multiple complications would be most practical and hence more feasible to implement. Considering that multimorbid obesity—that is, the occurrence of multiple complications in a single individual–is common^3^, the clinical scenario can be reframed as a multilabel classification problem. We handled this problem as separate binary classification tasks, building separate models for each outcome individually, as explained above. From each model, we extracted the top 20 clinically available features that were consistently selected: that is, with non-zero coefficients in the training set. In the optimization sets, after further pruning for correlated features (*r* > 0.6), we tested the feature importance of these features using the leave-one-out covariate importance procedure^49^, and we subsequently ranked these features by mean importance across all 18 outcomes and took forward the top 20. This set of shared features represented a core group of predictors that showed relevance across multiple conditions. We derived 18 separate models to predict each outcome, each retrained using these 20 features in the optimization sets and tested in the validation sets.

#### External validation

To test for generalizability of the shared OBSCORE model, we additionally performed external validation in the EPIC-Norfolk study^20,23^. EPIC-Norfolk is a prospective cohort study of more than 25,000 middle-aged individuals from the general population of Norfolk, England.

In the EPIC-Norfolk study, mortality was retrieved from the UK Office of National Statistics, as described before^20,21,23^. Hospital episode statistics and certificates of death were retrieved from the NHS digital database, with National Health Service numbers. Health records were coded by trained nosologists according to the International Statistical Classification of Diseases and Related Health Problems, 9th (ICD-9) or 10th Revision (ICD-10), and codes were merged owing to the long-term follow-up. Events were determined in case of disease as underlying cause of death or as the reason for hospitalization. To allow for comparisons, we used the same ICD-10/ICD-9 definitions for baseline disease exclusion and incident disease definitions, except for T2D (ICD-9: 250, ICD-10: E10–E14) and stroke (ICD-9: 433–435, ICD-10: I63, I65, I66). For external validation, we included 2,112 individuals from the EPIC-Norfolk cohort with a BMI ≥ 27 and with complete data on predictor variables (18 out of 20 variables were available, except for cystatin C and joint pain). We conducted a complete case analysis. The variables underwent the same standardization processes as in the UKB, including *Z*-score transformation of continuous variables and logarithmic transformation of skewed variables. We were able to externally validate 14 outcomes with more than 20 incident cases during a 10-year follow-up: angina pectoris (155 cases), arthropathy (122), cholelithiasis (48), coronary atherosclerosis (143), cardiovascular death (94), diaphragmatic hernia (128), GERD (78), hypercholesterolemia (60), hypertension (112), ischemic heart disease (217), MACE (109), myocardial infarction (106), stroke (59) and T2D (114). We used coefficients (model weights) from the OBSCORE model trained with 18 available predictors and tested performance in external validation: that is, without retraining the models. We evaluated performance using C-indices in analogy to UKB analyses, calculated over 1,000 bootstrap samples. We excluded prevalent cases based on diagnoses or early incident cases during follow-up (within the first 6 months).

To further assess generalizability across ancestries, we performed external validation for incident T2D in the G&H study, a population at high risk for T2D and with sufficient incident events to support reliable analyses for this outcome. G&H is a prospective study enrolling individuals from self-reported British Pakistani and British Bangladeshi backgrounds and aged 16 or over since 2015. We curated and harmonized routine UK NHS EHR data from primary care and secondary care and death information from the Office for National Statistics^50^. We extracted first occurrences of diagnoses and closest quantitative measures from EHRs to study baselines. T2D was ascertained by EHR-based diagnoses as described before^51^, and we used HbA1c > 48 mmol mol^−1^ to additionally exclude prevalent undiagnosed cases. A total of 1,740 individuals with complete data on 15 OBSCORE features, with a South Asian BMI cut-off of ≥22 kg m^−^^2^, which corresponds to a BMI of ~27 kg m^−^^2^ in white populations, as informed by ref. ^52^, and without prevalent T2D at baseline (study enrollment), were included for external validation. A total of 337 cases occurred during a median follow-up of 6.3 years, excluding incident cases within occurring within 6 months after baseline. We used coefficients from the OBSCORE model trained with the 15 available features (excluding urate, cystatin C, family history of heart disease, joint and non-specific chest pain) in the UKB for external validation.

#### Performance comparison with established models

We compared this model with the best-performing outcome-specific models—a model containing age, sex and BMI—and to features of other established classifiers of obesity: for example, metabolically healthy obesity (age, sex and binary variable based on systolic blood pressure < 130 mmHg and no antihypertensive medication, waist-to-hip ratio < 0.95 for women or < 1.03 for men and no prevalent T2D) as well as the SCORE2 model (age, sex, smoking status, total cholesterol, high-density lipoprotein cholesterol, systolic blood pressure) and ASCVD risk score (age, sex, smoking status, ancestry, systolic blood pressure, diastolic blood pressure, total cholesterol, high-density lipoprotein cholesterol, low-density lipoprotein cholesterol, T2D, antihypertensive medication, cholesterol-lowering medication, acetylsalicylic acid intake)^35,53,54^.

#### Calculation of performance metrics

To test how the shared clinical model performs at risk stratification, we computed the linear predictors for each outcome, resembling individual risk assigned by the prognostic model. We categorized the estimated risk by 20% (quintiles) of individuals for each outcome and calculated and visualized the actual observed 10-year event rate for each 20% group. We calculated risk ratios between the highest 20% and the lowest 20%. Moreover, we calculated rate ratios between the top 50% higher and lower risk groups and compared them to the rate ratios of individuals with obesity versus overweight. Using the estimated risk, we computed additional performance parameters including detection rates across FPRs ranging from 5% to 40%. FPRs were calculated as $\mathrm{FPR}=\frac{\mathrm{FP}}{\mathrm{TN}+\mathrm{FP}}$, where TN is true negatives; and detection rates (DRs) were calculated as $\mathrm{DR}=\frac{\mathrm{TP}}{\mathrm{FN}+\mathrm{TP}}$, where TP is true positives and FN is false negatives. Likelihood ratios (LRs) were computed as $\mathrm{LR}=\frac{\mathrm{DR}}{\mathrm{FPR}}$. To assess the real-world translational potential of the models, we calculated pre-test and post-test probabilities. Pre-test probabilities were derived from cumulative complication incidence in the study, and pre-test odds were calculated as $\mathrm{pretest}\,\mathrm{odds}=\frac{\mathrm{pretest}\,\mathrm{probability}}{\left(1-\mathrm{pretest}\,\mathrm{probability}\right)}$. Post-test probabilities at 10% FPR were calculated as $\mathrm{post}-\mathrm{test}\,\mathrm{probability}=\frac{(\mathrm{pretest}\,\mathrm{odds}\,\times \,\mathrm{LR})}{1+(\mathrm{pretest}\,\mathrm{odds}\,\times \,\mathrm{LR})}$. We assessed calibration visually with calibration plots and by calculating ratios and differences between estimated and observed risks (from Kaplan–Meier estimates) in the held-out validation set.

#### OBSCORE integration in the randomized controlled SURMOUNT-1 trial

SURMOUNT-1 is a randomized controlled trial of participants with BMI ≥30 kg m^−^^2^ or ≥27 kg m^−^^2^ with at least one weight-related comorbidity treated with tirzepatide (5 mg, 10 mg, 15 mg) versus placebo (NCT04184622)^7^. We applied the OBSCORE risk model to individuals participating in SURMOUNT-1. A total of 12 features of OBSCORE (age, sex, waist-to-height ratio, hypertension, total cholesterol, high-density lipoprotein cholesterol, HbA1c, alanine aminotransferase, creatinine, cystatin C, urate, smoking status) were available at baseline and at 72 weeks. Coefficients from models retrained with these 12 features were applied to participants from SURMOUNT-1 with available data (*n*_placebo_ = 388, *n*_TZP 5mg_ = 462, *n*_TZP 10mg_ = 479, *n*_TZP 15mg_ = 475) to calculate risk scores at baseline and at week 72. We first investigated the heterogeneity of treatment effects (body weight change, waist-to-height ratio change) across baseline predicted risk strata (quartiles) using linear regression models, whereby risk strata were coded numerically to test for linear trends. For each adiposity measure and risk score, we fit a model with treatment contrasts (placebo as reference). The model includes main effects for predicted risk strata and treatment arm, and their interaction term that tests whether treatment effects (versus placebo) change linearly across risk quartiles. EMMs for each treatment arm within each stratum were calculated using the emmeans package, with equal weight given to each stratum regardless of sample size. To test for changes in predicted risk from baseline to week 72 across all 18 outcomes, a one-way analysis of variance across treatment arms was employed, followed by unadjusted pairwise contrasts of the group means.

#### Subgroup and sensitivity analyses

To investigate performance of OBSCORE in different subgroups, we evaluated the performance of OBSCORE separately in individuals of genetically inferred non-European (*n* = 9,668) and European (sample size matched) ancestries as well as in individuals with the lower 50% and higher 50% of the Townsend deprivation index (median of the study population −2.05), without retraining in the subgroups. To test whether discriminative performance may be influenced by early incident cases that represent delayed diagnosis, we investigated C-indices of OBSCORE when excluding early incident cases additionally at 1 year, 1.5 years and 2 years after baseline for each outcome.

### Reporting summary

Further information on research design is available in the Nature Portfolio Reporting Summary linked to this article.

## Online content

Any methods, additional references, Nature Portfolio reporting summaries, source data, extended data, supplementary information, acknowledgements, peer review information; details of author contributions and competing interests; and statements of data and code availability are available at 10.1038/s41591-026-04353-2.

## Supplementary information

Supplementary Information Supplementary Figs. 1–5, and the Genes & Health Research Team.

Reporting Summary

Supplementary Tables Supplementary Tables 1–11

## Source data

Source Data Figs. 1–6 and Extended Data Figs. 1–9 Source data.

## Figures and Tables

**Extended Data Table 1:**
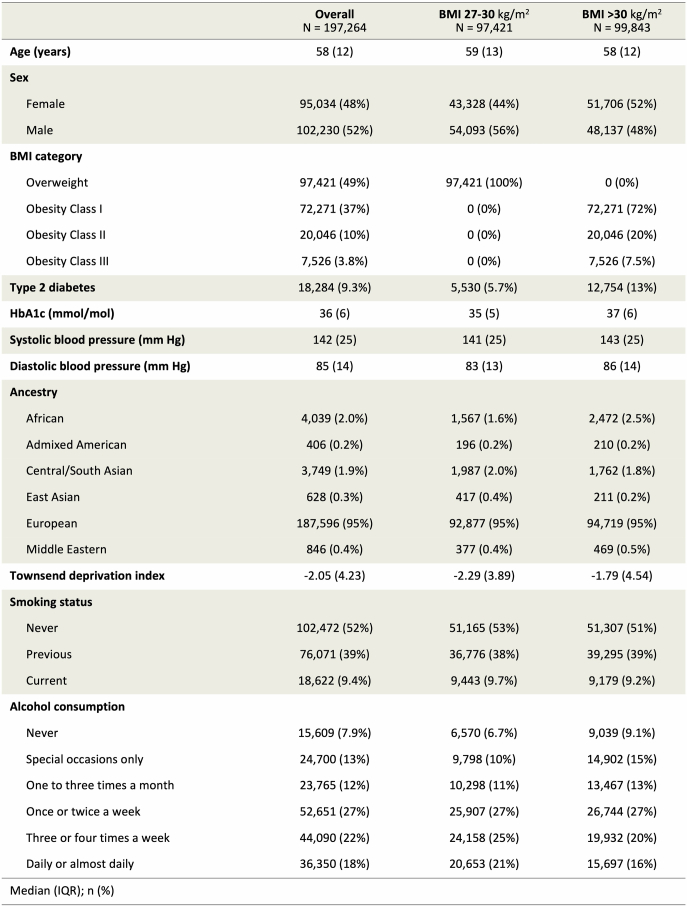
Study participant characteristics

## Data Availability

All individual-level data are publicly available to bona fide researchers upon application from the UKB (https://www.ukbiobank.ac.uk/), EPIC-Norfolk (https://www.epic-norfolk.org.uk/for-researchers/data-sharing/data-requests/) and G&H (https://www.genesandhealth.org/). SURMOUNT-1: Lilly provides access to all individual participant data collected during the trial, after anonymization, with the exception of pharmacokinetic or genetic data. Data are available on request 6 months after the indication studied has been approved in the USA and EU and after primary publication acceptance, whichever is later. No expiration date of data requests is currently set once data are made available. Access is provided after a proposal has been approved by an independent review committee identified for this purpose and after receipt of a signed data sharing agreement. Data and documents, including the study protocol, statistical analysis plan, clinical study report, blank or annotated case report forms, will be provided in a secure data sharing environment. For details on submitting a request, see the instructions provided at www.vivli.org. Source data are provided with this paper.

## Code availability

Statistical code is available at https://github.com/comp-med/obesity-prognosis.
