# Supplementary material for: Data-driven prioritization of high-risk individuals for weight loss interventions
Source: Nat Med. 2026 Apr 30;32(6):2117–27. doi: 10.1038/s41591-026-04353-2 (PMC13279260; doi:10.1038/s41591-026-04353-2)
Supplement: Supplementary file 1 — Supplementary Figs. 1–5, and the Genes & Health Research Team. [file 41591_2026_4353_MOESM1_ESM.pdf]

nature medicine

Supplementary information https://doi.org/10.1038/s41591-026-04353-2

Data-driven prioritization of high-risk individuals for weight loss interventions

In the format provided by the authors and unedited

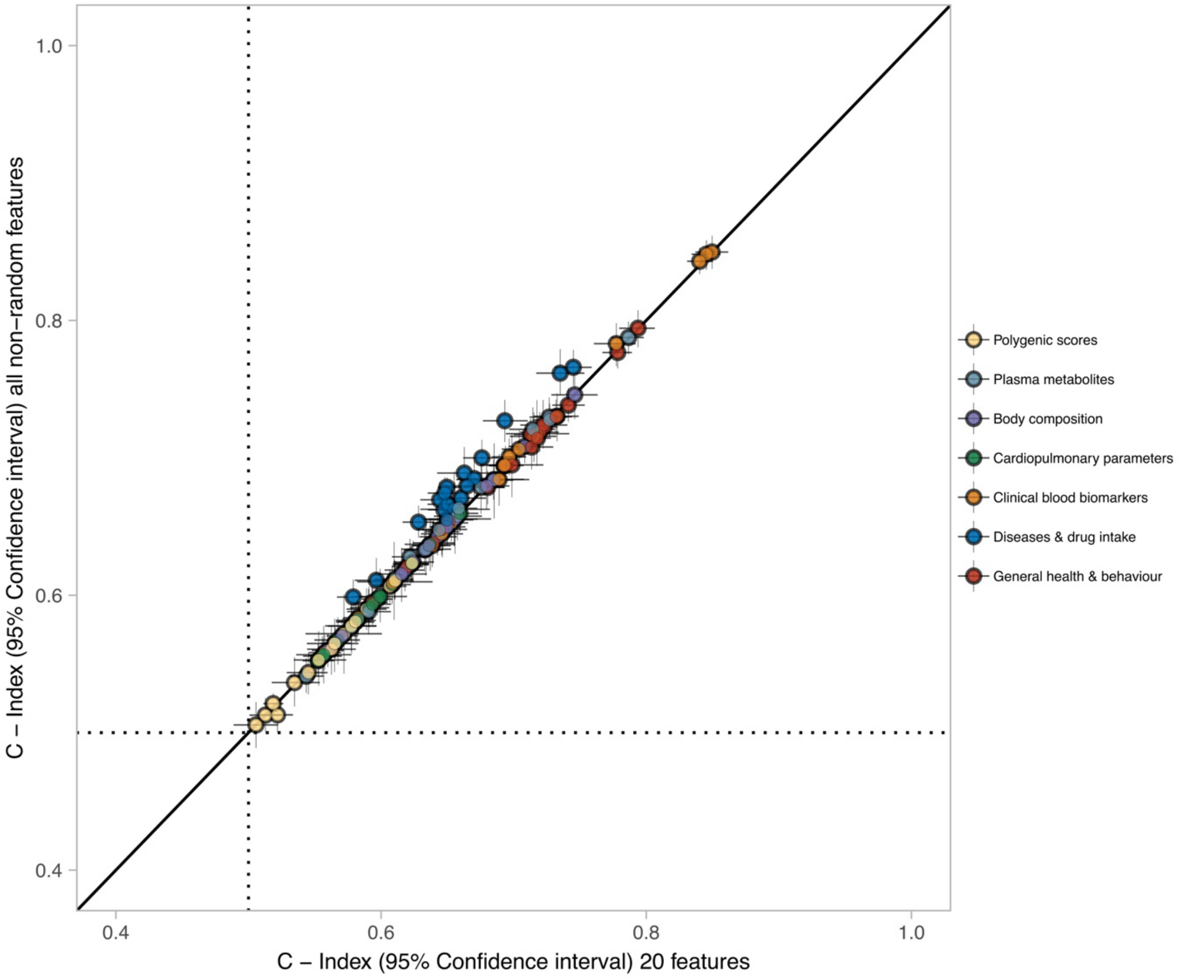

Supplementary Figure 1. Unimodal outcome-specific models with up to 20 features and with all non-random features. The performances (depicted as Concordance index (C-index) with 95% confidence intervals estimated from 1,000 bootstrap resamples) of unimodal models with up to 20 features (X-axis) are compared with performances of unimodal models with all non-randomly selected features (Y-axis). Sample sizes are display in Supplementary Table 2.

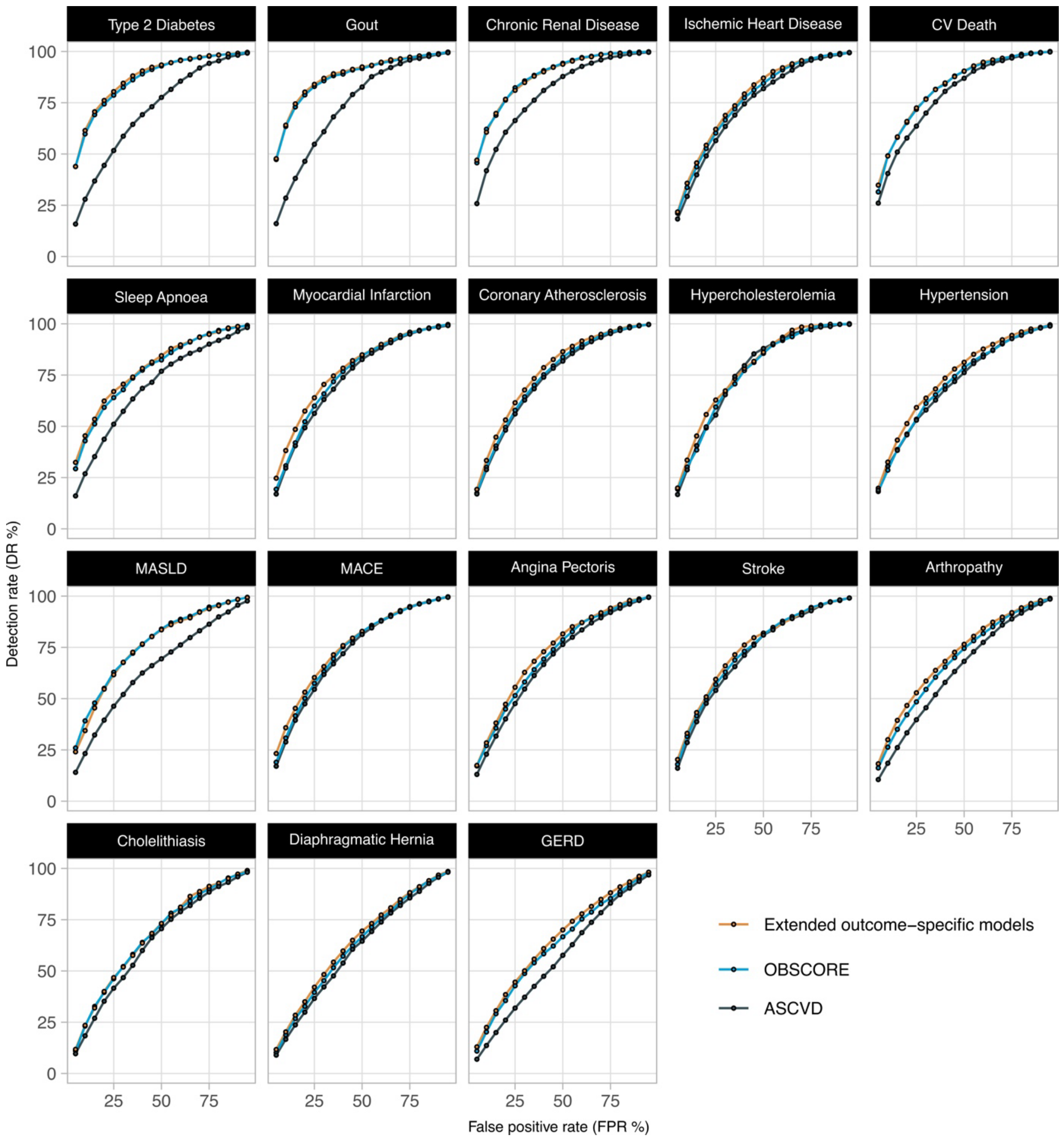

Supplementary Figure 2. Detection rates (DR) across different false positive rate (FPR) thresholds. The DR corresponds to the proportion of true cases identified (sensitivity), while FPR corresponds to the proportion of non-cases incorrectly classified as events (1-specificity). Each curve illustrates the trade-of between sensitivity and specificity for the three risk models compared. For example, at a FPR of 10% (i.e., accepting that 10% of individuals without the outcome will be incorrectly flagged as high risk), the OBSCORE detected 60% of individuals with incident T2D within 10 years, compared to 28% by ASCVD and 61% by the extended outcome-specific model.

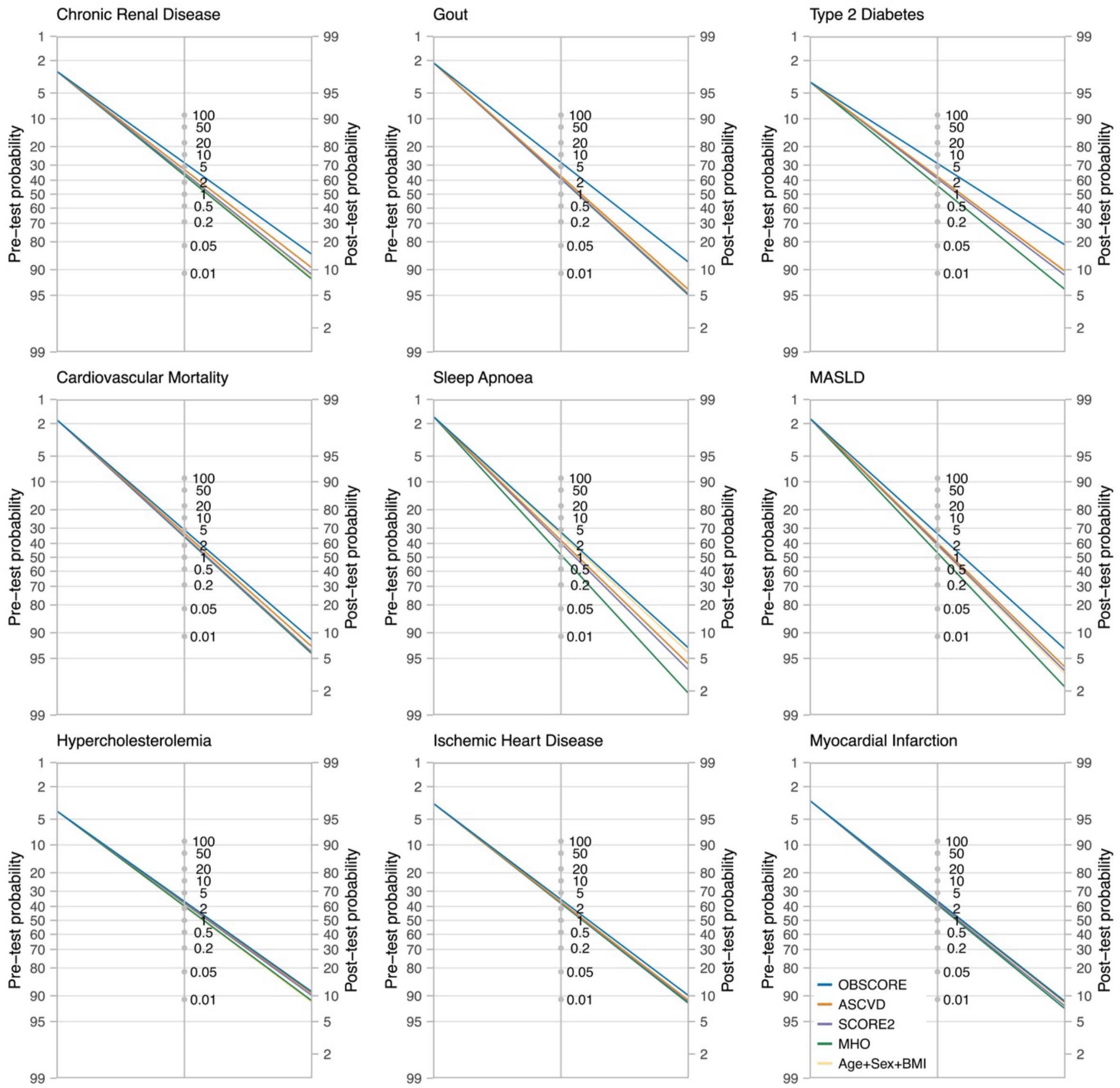

Supplementary Figure 3. Fagan's nomograms for different outcomes displaying pre- and post-test probabilities using different models. Pre-test probability is calculated based on incidence in the study, and post-test probability is calculated based on likelihood ratio for each model.

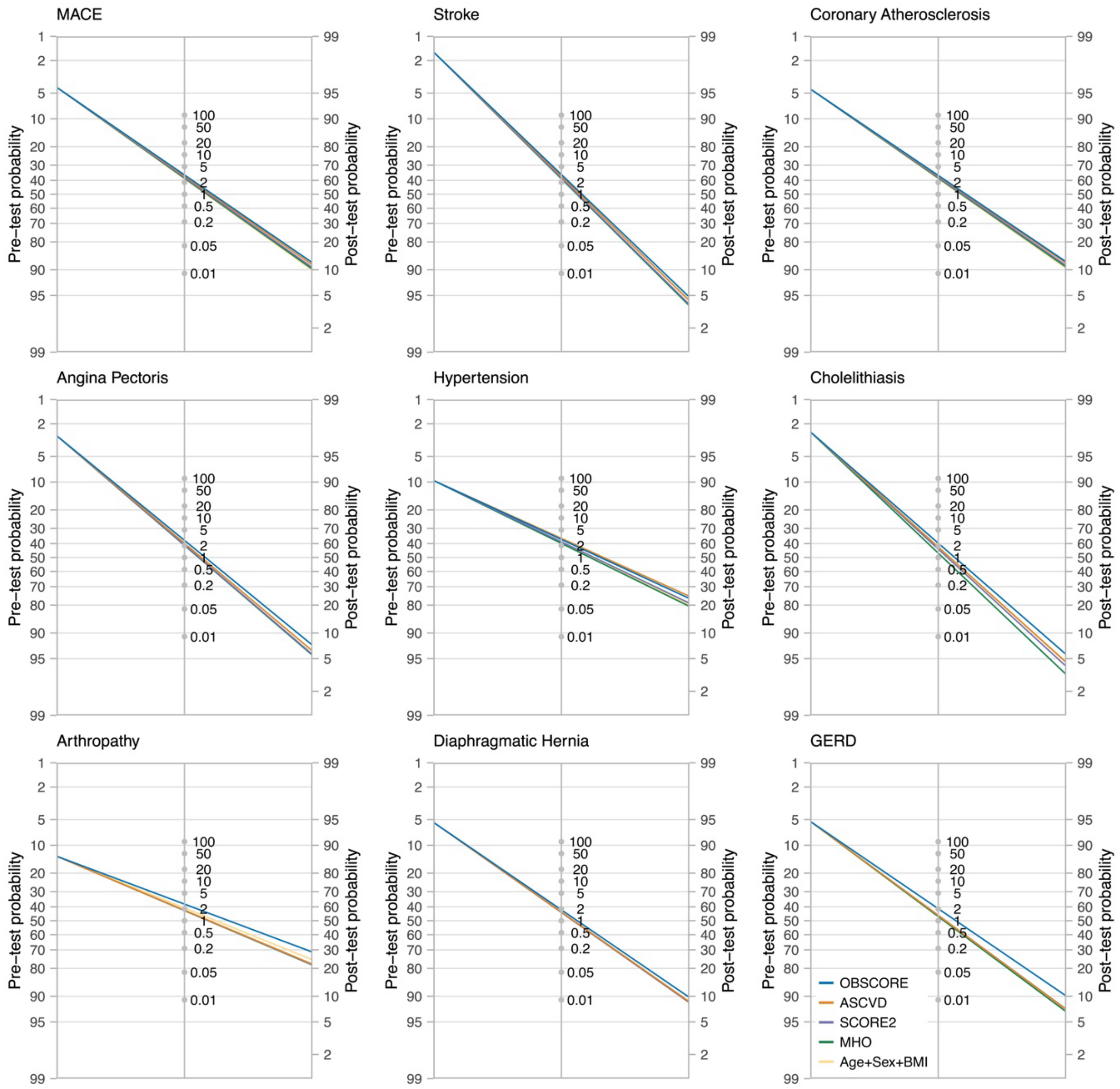

Supplementary Figure 4. Fagan's nomograms for different outcomes displaying pre- and post-test probabilities using different models. Pre-test probability is calculated based on incidence in the study, and post-test probability is calculated based on likelihood ratio for each model.

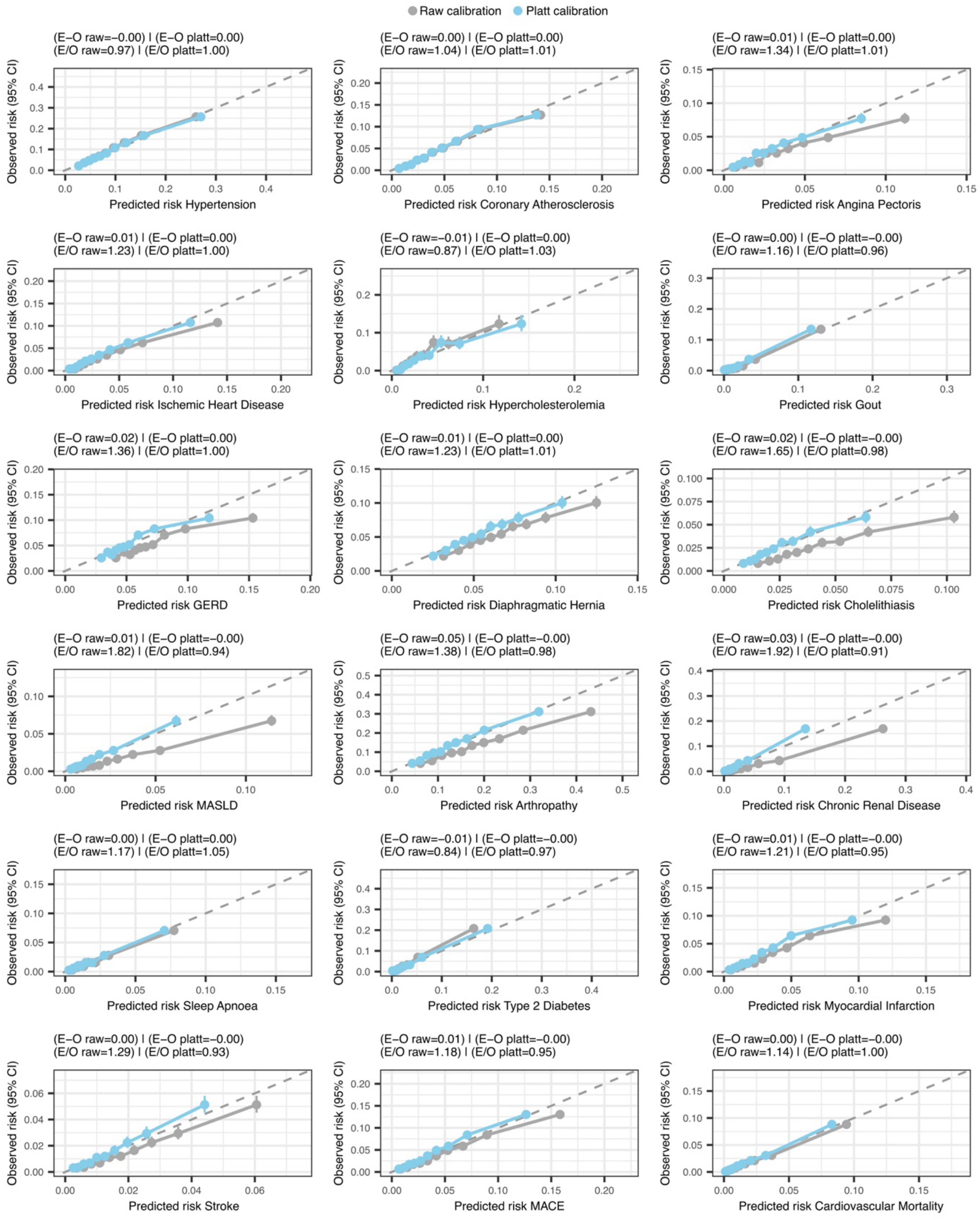

Supplementary Figure 5. Calibration plots. Calibration of OBSCORE across outcomes as tested in the held-out validation sets. Grey lines depict direct out-of-the-box calibrations and blue lines depict Platt calibration across bins of deciles of risk. E-O = Estimated-Observed risk difference; E/O = Estimated/Observed risk ratio. Sample sizes are displayed in Supplementary Table 2.

Genes & Health Research Team

Eamonn Maher	Aston University
Shabana Chaudhary	Blizard Institute, Queen Mary University of London
Joseph Gafton	Blizard Institute, Queen Mary University of London
Karen A Hunt	Blizard Institute, Queen Mary University of London
Shapna Hussain	Blizard Institute, Queen Mary University of London
Kamrul Islam	Blizard Institute, Queen Mary University of London
Mohammed Bodrul Mazid	Blizard Institute, Queen Mary University of London
Elizabeth Owor	Blizard Institute, Queen Mary University of London
Jessry Russell	Blizard Institute, Queen Mary University of London
Nishat Safa	Blizard Institute, Queen Mary University of London
John Solly	Blizard Institute, Queen Mary University of London
Marie Spreckley	Blizard Institute, Queen Mary University of London
David A Van Heel	Blizard Institute, Queen Mary University of London
Jan Whalley	Blizard Institute, Queen Mary University of London
Ishevanhu Zengeya	Blizard Institute, Queen Mary University of London
Emily Mantle	Blizard Institute, Queen Mary University of London
Shaheen Akhtar	Bradford Teaching Hospitals NHS Foundation Trust
Samina Ashraf	Bradford Teaching Hospitals NHS Foundation Trust
Dan Mason	Bradford Teaching Hospitals NHS Foundation Trust
John Wright	Bradford Teaching Hospitals NHS Foundation Trust
Daniel MacArthur	Garvan Institute
Michael Simpson	King's College London
Richard C Trembath	King's College London
Gerome Breen	Kings College London
Raymond Chung	Kings College London
Sang Hyuck Lee	Kings College London
Omar Asgar	Manchester University Hospitals
Joanne Harvey	Manchester University Hospitals
Karen Tricker	Manchester University Hospitals
Caroline Winckley	Manchester University Hospitals
Hanifa Khatun	Manchester University Hospitals
Amna Asif	Manchester University Hospitals
Claudia Langenberg	Precision Healthcare University Research Institute, Queen Mary University of London
Grainne Colligan	Social Action for Health (charity)
Ceri Durham	Social Action for Health (charity)
Bill Newman	University of Manchester
Ahsan Khan	Waltham Forest Council
Hilary Martin	Wellcome Sanger Institute
Teng Heng	Wellcome Sanger Institute
Matt Hurles	Wellcome Sanger Institute
Vivek Iyer	Wellcome Sanger Institute

Georgios Kalantzis	Wellcome Sanger Institute
Vladimir Ovchinnikov	Wellcome Sanger Institute
Iaroslav Popov	Wellcome Sanger Institute
Klaudia Walter	Wellcome Sanger Institute
Panos Deloukas	William Harvey Research Institute, Queen Mary University of London
David Collier	William Harvey Research Institute, Queen Mary Universityof London
Ana Angel	Wolfson Institute of Population Health, Queen Mary University of London
Saeed Bidi	Wolfson Institute of Population Health, Queen Mary University of London
Fabiola Eto	Wolfson Institute of Population Health, Queen Mary University of London
Sarah Finer	Wolfson Institute of Population Health, Queen Mary University of London
Chris Griffiths	Wolfson Institute of Population Health, Queen Mary University of London
Sam Hodgson	Wolfson Institute of Population Health, Queen Mary University of London
Benjamin M Jacobs	Wolfson Institute of Population Health, Queen Mary University of London
Rohini Mathur	Wolfson Institute of Population Health, Queen Mary University of London
Caroline Morton	Wolfson Institute of Population Health, Queen Mary University of London
Asma Qureshi	Wolfson Institute of Population Health, Queen Mary University of London
Stuart Rison	Wolfson Institute of Population Health, Queen Mary University of London
Annum Salman	Wolfson Institute of Population Health, Queen Mary University of London
Miriam Samuel	Wolfson Institute of Population Health, Queen Mary University of London
Moneeza K Siddiqui	Wolfson Institute of Population Health, Queen Mary University of London
Daniel Stow	Wolfson Institute of Population Health, Queen Mary University of London
Sabina Yasmin	Wolfson Institute of Population Health, Queen Mary University of London
Julia Zöllner	Wolfson Institute of Population Health, Queen Mary University of London
Sheik Dowlut	Wolfson Institute of Population Health, Queen Mary University of London
