# Supplementary material for: Data-driven prioritization of high-risk individuals for weight loss interventions
Source: Nat Med. 2026 Apr 30;32(6):2117–27. doi: 10.1038/s41591-026-04353-2 (PMC13279260; doi:10.1038/s41591-026-04353-2)
Supplement: Supplementary file 2 — Reporting Summary [file 41591_2026_4353_MOESM2_ESM.pdf]

nature portfolio

Corresponding author(s): Claudia Langenberg

Last updated by author(s): 25.02.2026

Reporting Summary

Nature Portfolio wishes to improve the reproducibility of the work that we publish. This form provides structure for consistency and transparency in reporting. For further information on Nature Portfolio policies, see our Editorial Policies and the Editorial Policy Checklist.

Statistics

For all statistical analyses, confirm that the following items are present in the figure legend, table legend, main text, or Methods section.

n/a	Confirmed	
☐	☒	The exact sample size (*n*) for each experimental group/condition, given as a discrete number and unit of measurement
☐	☒	A statement on whether measurements were taken from distinct samples or whether the same sample was measured repeatedly
☐	☒	The statistical test(s) used AND whether they are one- or two-sided<br>*Only common tests should be described solely by name; describe more complex techniques in the Methods section.*
☐	☒	A description of all covariates tested
☐	☒	A description of any assumptions or corrections, such as tests of normality and adjustment for multiple comparisons
☐	☒	A full description of the statistical parameters including central tendency (e.g. means) or other basic estimates (e.g. regression coefficient) AND variation (e.g. standard deviation) or associated estimates of uncertainty (e.g. confidence intervals)
☐	☒	For null hypothesis testing, the test statistic (e.g. *F*, *t*, *r*) with confidence intervals, effect sizes, degrees of freedom and *P* value noted<br>*Give P values as exact values whenever suitable.*
☒	☐	For Bayesian analysis, information on the choice of priors and Markov chain Monte Carlo settings
☒	☐	For hierarchical and complex designs, identification of the appropriate level for tests and full reporting of outcomes
☐	☒	Estimates of effect sizes (e.g. Cohen's *d*, Pearson's *r*), indicating how they were calculated

Our web collection on statistics for biologists contains articles on many of the points above.

Software and code

Policy information about availability of computer code

Data collection	No software was used for data collection.
Data analysis	Data analyses were performed in R (version 4.3.1.), using following packages arrow (16.1.0), caret (6.0-94), corrplot (0.95), data.table (1.17.0), doMC (1.3.8), doParallel (1.0.17), dplyr (1.1.4), fastDummies (1.7.5), flextable (0.9.6), foreach (1.5.2), ggplot2 (3.5.1), ggradar (0.2), ggrepel (0.9.5), ggridges (0.5.6), ggsci (3.2.0), glmnet (4.1-8), gtsummary (2.0.3), igraph (2.1.4), influential (2.2.9), magrittr (2.0.3), miceRanger (1.5.0), patchwork (1.3.0), Rfast (2.1.0), ROSE (0.0-4), shiny (1.9.1), shinyBS (0.61.1), shiny themes (1.2.0), sna (2.8), survival (3.8-3), survminer (0.4.9), tidyr (1.3.1), tidyverse (2.0.0). Analysis code is available on https://github.com/comp-med/obesity-prognosis.

For manuscripts utilizing custom algorithms or software that are central to the research but not yet described in published literature, software must be made available to editors and reviewers. We strongly encourage code deposition in a community repository (e.g. GitHub). See the Nature Portfolio guidelines for submitting code & software for further information.

Data

Policy information about availability of data

All manuscripts must include a data availability statement. This statement should provide the following information, where applicable:

- Accession codes, unique identifiers, or web links for publicly available datasets
- A description of any restrictions on data availability
- For clinical datasets or third party data, please ensure that the statement adheres to our policy

All individual-level data is publicly available on application to bona fide researchers from the UKB (https://www.ukbiobank.ac.uk/), EPIC-Norfolk (https://www.epic-norfolk.org.uk/for-researchers/data-sharing/data-requests/), and Genes & Health (https://www.genesandhealth.org/). For UKB, this research has been conducted under the applications 44448 and 30418. SURMOUNT-1: Lilly provides access to all individual participant data collected during the trial, after anonymization, with the exception of pharmacokinetic or genetic data. Data are available on request 6 months after the indication studied has been approved in the US and EU and after primary publication acceptance, whichever is later. No expiration date of data requests is currently set once data are made available. Access is provided after a proposal has been approved by an independent review committee identified for this purpose and after receipt of a signed data sharing agreement. Data and documents, including the study protocol, statistical analysis plan, clinical study report, blank or annotated case report forms, will be provided in a secure data sharing environment. For details on submitting a request, see the instructions provided at www.vivli.org.

Research involving human participants, their data, or biological material

Policy information about studies with human participants or human data. See also policy information about sex, gender (identity/presentation), and sexual orientation and race, ethnicity and racism.

Reporting on sex and gender	In UKB We defined 'female' and 'male' sex including participants where the recorded sex and sex chromosomes aligned (XX for females and XY for males). The recorded sex was self-reported, and it was not possible to distinguish sex from gender. We acknowledge the importance of distinguishing between sex and gender in research and that chromosomal make-up does not always align with self-identified gender.
Reporting on race, ethnicity, or other socially relevant groupings	We used previously published ancestral assignments by the pan-UKB consortium to assign individuals to ancestral groups, and made a further effort to assign unclassified individuals to their respective ancestries based on a k-nearest neighbour approach using genetic principal components.
Population characteristics	UK Biobank is a prospective cohort study from the UK that contains more than 500,000 volunteers between 40 and 69 years of age at inclusion. The cohort has been extensively described elsewhere (www.ukbiobank.ac.uk). We included 197,264 individuals based on criteria of recent weight-loss medication trials, e.g. excluding BMI <27 kg/m^2, pregnancy, and others.
Recruitment	All individuals between the age of 40-69 (men and women) who were registered with the National Health Service and living within a 25-mile radius from one of 22 recruitment centers spread across the United Kingdom were invited to participate in 2006-2010. Overall, about 9.2M individuals were invited to recruit around 0.5M individuals.
Ethics oversight	UKB was granted ethical approval from the North West Centre for Research Ethics Committee (11/NW/0382), in the form of a Research Tissue Bank (RTB) approval. All participants provided informed consent, and study procedures were performed according to the Helsinki declaration. This study was conducted with data accessed under the application numbers 44448 and 30418. The EPIC-Norfolk study has been approved by the Norfolk Research Ethics Committee, under the reference number 05/Q0101/191. G&H was approved by the London Southeast NRES Committee of the Health Research Authority (14/LO/1240). SURMOUNT-1 was conducted according to the principles of the Declaration of Helsinki and Good Clinical Practice guidelines and was approved by independent ethics committees or institutional review boards at each of the 119 sites across nine countries.

Note that full information on the approval of the study protocol must also be provided in the manuscript.

Field-specific reporting

Please select the one below that is the best fit for your research. If you are not sure, read the appropriate sections before making your selection.

☒ Life sciences ☐ Behavioural & social sciences ☐ Ecological, evolutionary & environmental sciences

For a reference copy of the document with all sections, see nature.com/documents/nr-reporting-summary-flat.pdf

Life sciences study design

All studies must disclose on these points even when the disclosure is negative.

Sample size	UKB: We included 197,264 individuals with BMI>=27 kg/m^2<br>EPIC-Norfolk: We included 2,112 individuals with complete data on available OBSCORE features.<br>Genes & Health: We included 1,740 individuals with complete data on available OBSCORE features.<br>SURMOUNT-1: We included up to 1,804 individuals (placebo= 388, TZP 5mg = 462, TZP 10mg = 479, TZP 15mg = 475). This was an exploratory, non-prespecified analysis of individual level data in SURMOUNT-1.
Data exclusions	UKB: We excluded individuals based on criteria of recent weight-loss medication trials, e.g. BMI <27 kg/m^2, pregnancy, and others.

Data exclusions	Moreover, we excluded individuals where we did not have access to matching genotyping (array or sequencing-based) and polygenic score data. Furthermore, individual samples failing standard genotyping quality control or not assigned to one of the three major ancestral groups were excluded. These decisions were made before performing any statistical analysis. Participants were also excluded based on BMI<27 kg/m^2 in EPIC-Norfolk and Genes & Health.
Replication	UKB: We divided the dataset into test, optimisation and validation sets to derive the model and test (replicate) the models in internal validation. External validation: Additionally, to display generalisability of the OBSCORE model, we performed external validation in a non-overlapping external study, European Investigation into Cancer - Norfolk (EPIC-Norfolk) study. We were able to validate performance and generalisability for 14 out of 18 outcomes utilising 18 of the 20 features included in OBSCORE. The validation study displayed high generalisability and performance of the model in an external study. We were able to test performance and generalisability of OBSCORE for incident T2D additionally in the Genes & Health study, which is exclusively enrolling individuals with self-reported Bangladeshi or Pakistani backgrounds.
Randomization	UKB/EPIC-Norfolk/Genes & Health: N/A.<br>SURMOUNT-1: Participants were randomly assigned in a 1:1:1:1 ratio to receive tirzepatide at a dose of 5 mg, 10 mg, 15 mg, or placebo.
Blinding	UKB/EPIC-Norfolk/Genes & Health: N/A.<br>SURMOUNT-1: Double blinded in the original study, no blinding during these analyses.

Reporting for specific materials, systems and methods

We require information from authors about some types of materials, experimental systems and methods used in many studies. Here, indicate whether each material, system or method listed is relevant to your study. If you are not sure if a list item applies to your research, read the appropriate section before selecting a response.

Materials & experimental systems

n/a	Involved in the study
☒	☐ Antibodies
☒	☐ Eukaryotic cell lines
☒	☐ Palaeontology and archaeology
☒	☐ Animals and other organisms
☐	☒ Clinical data
☒	☐ Dual use research of concern
☒	☐ Plants

Methods

n/a	Involved in the study
☒	☐ ChIP-seq
☒	☐ Flow cytometry
☒	☐ MRI-based neuroimaging

Clinical data

Policy information about clinical studies
All manuscripts should comply with the ICMJE guidelines for publication of clinical research and a completed CONSORT checklist must be included with all submissions.

Clinical trial registration	NCT04184622
Study protocol	See protocol for: Jastreboff AM, Aronne LJ, Ahmad NN, et al. Tirzepatide once weekly for the treatment of obesity. N Engl J Med 2022;387:205-16. (https://www.nejm.org/doi/suppl/10.1056/NEJMoa2206038/suppl_file/nejmoa2206038_protocol.pdf)
Data collection	Described in Jastreboff AM, Aronne LJ, Ahmad NN, et al. Tirzepatide once weekly for the treatment of obesity. N Engl J Med 2022;387:205-16.
Outcomes	We performed a non-prespecified exploratory analysis of the SURMOUNT-1 trial.

Plants

Seed stocks	*Report on the source of all seed stocks or other plant material used. If applicable, state the seed stock centre and catalogue number. If plant specimens were collected from the field, describe the collection location, date and sampling procedures.*
Novel plant genotypes	*Describe the methods by which all novel plant genotypes were produced. This includes those generated by transgenic approaches, gene editing, chemical/radiation-based mutagenesis and hybridization. For transgenic lines, describe the transformation method, the number of independent lines analyzed and the generation upon which experiments were performed. For gene-edited lines, describe the editor used, the endogenous sequence targeted for editing, the targeting guide RNA sequence (if applicable) and how the editor was applied.*
Authentication	*Describe any authentication procedures for each seed stock used or novel genotype generated. Describe any experiments used to assess the effect of a mutation and, where applicable, how potential secondary effects (e.g. second site T-DNA insertions, mosiacism, off-target gene editing) were examined.*
